# Study on tumor ecology and precision medicine strategy of oral squamous cell carcinoma based on multi-omics cell atlas

**DOI:** 10.3389/fimmu.2026.1925952

**Published:** 2026-10-09

**Authors:** Qingkang Meng, Diwei Huo, Fei Xu, Naiqiang Liu, Muchen Fan, Yewen Wu, Xiujie Chen

**Affiliations:** 1 The First Affiliated Hospital of Wannan Medical University, Yijishan Hospital of Wannan Medical University, Wuhu, China; 2 College of Bioinformatics Science and Technology, Harbin Medical University, Harbin, China; 3 The Second Affiliated Hospital of Harbin Medical University, Harbin, China

**Keywords:** multi-omics, oral squamous cell carcinoma, precision medicine, tumor ecology, tumor heterogeneity

## Abstract

**Introduction:**

Oral squamous cell carcinoma (OSCC) is characterized by inter- and intratumoral heterogeneity, which complicates patient stratification and treatment. Although single-cell studies have improved the resolution of the OSCC microenvironment, the relationships among cellular states, spatial organization, immune-repertoire dynamics, and patient-level ecosystem patterns remain incompletely understood.

**Methods:**

We integrated single-cell RNA sequencing, single-cell T-cell receptor sequencing, spatial transcriptomics, and bulk RNA-sequencing data to characterize OSCC.

**Results:**

We constructed a multi-omics cellular atlas comprising 88,442 cells and 63 cell subclusters, revealing complementary malignant-cell heterogeneity at the levels of transcriptional subclusters and recurrent meta-programs. Spatial and cell-cell communication analysis identified a reproducible association between PI3+ cancer-associated fibroblasts and APC_Myeloid cells and supported a candidate collagen-associated communication axis within tumor niches. Integration of paired transcriptomic and TCR data delineated a CD8+ T-cell pseudotime continuum accompanied by increasing clonal expansion and exhaustion-associated features, with spatial analysis linking intermediate trajectory states to regulatory T-cell and inflammatory NKT-cell-rich tumor-front regions. Extending the single-cell atlas to bulk cohorts by deconvolution, we defined four OSCC ecotypes and four coordinated cell programs that captured patient-level ecosystem organization and showed distinct molecular and clinical associations. We developed exploratory models for prognosis, recurrence, and immunotherapy sensitivity and established the OSCC Atlas as an online resource for data exploration.

**Discussion:**

This integrative framework links cellular heterogeneity, spatial organization, immune dynamics, and patient-level ecology, providing testable hypotheses for the biological and clinical stratification of OSCC.

## Introduction

Oral squamous cell carcinoma (OSCC) is the most common type of head and neck squamous cell carcinoma (HNSCC), with a high incidence and poor prognosis (1, 2). Current treatments include surgery, chemotherapy, radiotherapy, or their combinations (3). Although surgical resection is the primary treatment for early-stage disease, 20%–40% of patients still experience local recurrence and/or lymph-node metastasis after surgery (4). Many patients are diagnosed at an advanced stage and require multimodal treatment, for which curative treatment is often difficult to achieve.

Since 2016, programed cell death protein 1 (PD-1) inhibitors have been approved for recurrent or metastatic HNSCC (5, 6), but the overall response rate remains approximately 20%. In clinical practice, immunotherapy eligibility is commonly assessed using the combined positive score (CPS). Although approximately 85% of OSCC patients have a CPS greater than 1, only a subset respond to pembrolizumab (3), indicating that CPS alone has limited ability to identify patients who will benefit from immunotherapy. Thus, despite advances in multimodal therapy and immunotherapy, long-term survival in OSCC has not improved substantially over recent decades (2, 7), highlighting the need for improved patient stratification and treatment-response biomarkers.

The complex intra-tumoral heterogeneity (ITH) of OSCC is one of the main reasons for its clinical treatment challenges (8). ITH increases the difficulty of early diagnosis and prognostic assessment of OSCC. Additionally, due to the presence of cell subclusters with different biological characteristics within tumor tissues (9), some may be inherently drug-resistant or become drug-resistant after treatment. The accumulation of residual drug-resistant subclusters can eventually lead to overall drug resistance and pose a significant risk for recurrence. Therefore, in-depth exploration of OSCC heterogeneity, particularly ITH, is crucial for advancing treatment strategies. To address HNSCC heterogeneity, Chung et al. first defined four molecular subtypes of HNSCC in 2004 based on gene expression patterns (7). Building on this, Walter et al. validated these subtypes in 2013, naming them basal, mesenchymal, atypical, and classical, respectively (8). Although these studies substantially advanced the molecular characterization of HNSCC, the resulting subtypes have remained predominantly descriptive, and their added value for patient stratification and treatment selection has not been fully established. Moreover, because these classifications were derived from bulk-level transcriptomic profiles, they provide limited resolution of the cellular heterogeneity underlying each molecular subtype.

The rapid development of multi-omics sequencing technology and its applications has revolutionized the understanding of tumor heterogeneity. Using single-cell RNA sequencing (scRNA-Seq), researchers can explore the ITH of OSCC in great detail, reconstruct the evolutionary paths of malignant OSCC cells, and identify potential drugs to block progression, thereby delaying or preventing further deterioration of OSCC (12). Compared to scRNA-Seq, spatial transcriptome sequencing (ST-Seq) not only reveals gene expression patterns but also preserves the spatial location of cells, offering a new spatial perspective. Using ST-Seq, Li et al. discovered that kidney cancer cells with high expression of epithelial-mesenchymal transition (EMT)-related genes are enriched at the tumor’s leading edge, highlighting the link between EMT and the invasive behavior of kidney cancer (9). However, current mainstream ST-Seq technologies (e.g., 10X Visium) do not achieve true single-cell resolution, so combining ST-Seq with scRNA-Seq enables high-resolution gene expression analysis while compensating for the loss of spatial information. This integration is particularly important for three-dimensional characterization of the tumor microenvironment (TME) and for understanding cellular interactions within it (10). T cells in TME exhibit extensive clonal expansion and an exhausted phenotype (11–16), with a notable relationship between these features (17). To analyze T cell clonal diversity and function at high resolution, single-cell TCR sequencing (scTCR-Seq) was developed (18). Using this method, researchers found that exhausted CD8+ T cells and regulatory T cells are enriched and exhibit significant clonal expansion in hepatocellular carcinoma (HCC), indicating an immunosuppressive TME (19). Overall, these high-resolution multi-omics sequencing technologies have greatly expanded our understanding of cancer. Additionally, studying cancers from multiple perspectives enhances the characterization of cellular features within the TME (20, 21), enabling a more precise and unbiased exploration of OSCC’s complex intra-tumor heterogeneity.

Recent studies conceptualize tumors as multicellular ecosystems in which malignant, stromal, and immune cells interact to shape tumor evolution, immune escape, and treatment response (22–29). Although Puram et al. (30) characterized HNSCC heterogeneity from an ecosystem perspective using scRNA-Seq, this analysis was limited to a single omics modality. A comprehensive multi-omics characterization of OSCC ecology remains lacking.

In this study, we integrated scRNA-Seq, scTCR-Seq, spatial transcriptomic, and bulk RNA-Seq datasets to characterize OSCC from a tumor-ecosystem perspective. We aimed to construct a multidimensional cellular atlas, examine malignant, stromal, and immune-cell heterogeneity and their spatial relationships, and investigate the dynamics of T-cell states. We further explored ecosystem-level patient stratification by defining OSCC ecotypes and coordinated cell programs and developed preliminary prediction models based on deconvolved cellular compositions. Collectively, these analysis were designed to provide an integrated framework for characterizing OSCC ecology and to generate testable hypotheses for future mechanistic and clinical validation.

## Results

### Cellular atlas construction and malignant-cell heterogeneity

To reveal the complex heterogeneity of OSCC at single-cell resolution, unsupervised clustering was performed to identify the major cell types in OSCC ecology (**Figure 1A**). After quality control, scRNA-Seq data from 114,148 cells across 41 OSCC patients were integrated. Based on batch effect-corrected expression profiles (**Supplementary Figure S1A, B**) and the optimal PCs after dimensionality reduction (**Supplementary Figure S1C, D**), 28 cell clusters were created. All cell clusters were thoroughly annotated as 11 major cell types, including B cells, CD4+ T cells, CD8+ T cells, dendritic cells, endothelial cells, epithelial cells, fibroblasts, macrophages, mast cells, myocytes, and plasma cells (**Figures 1B, C**, **Supplementary Table S1**). Compared with the reference endothelial cells, epithelial cell clusters 1, 5, 11, 13, 16, 23, 26, and 28 exhibited significantly higher CNV scores (Wilcoxon test, P<0.0001 for all comparisons; **Figure 1D**). Thus, these epithelial cell clusters were classified as putative malignant cell clusters, containing 36,879 cells (**Figure 1E**).

**Figure 1:**
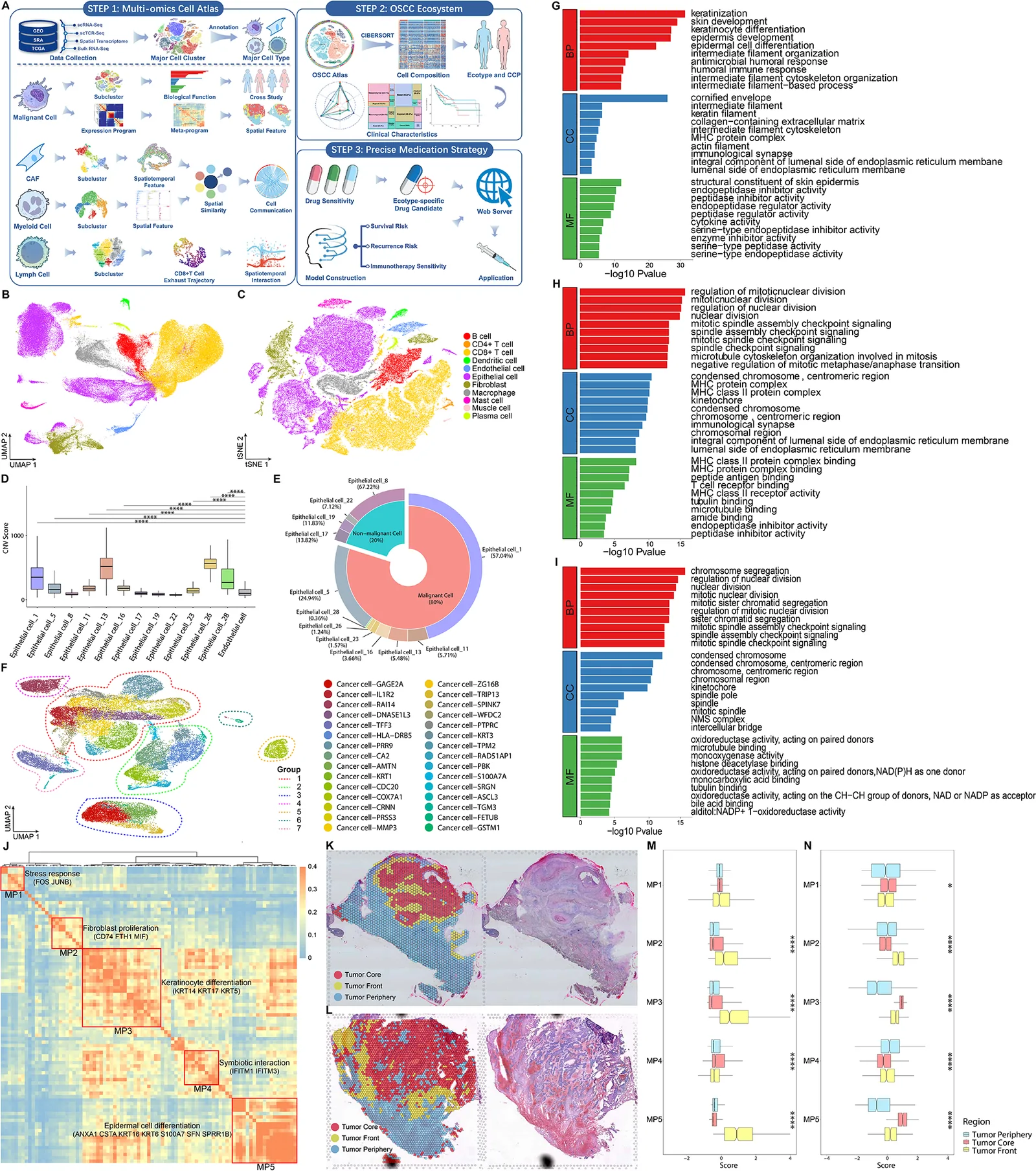
**(A)** Overview of the study. **(B)** UMAP dimensionality reduction mapping plot of cell cluster annotation result. **(C)** t-SNE dimensionality reduction mapping plot of cell cluster annotation result. **(D)** Box plot of CNV score. **(E)** Pie chart of the proportion of epithelial cell clusters. **(F)** UMAP dimensionality reduction mapping plot of malignant cell subclusters. **(G)** GO enrichment analysis bar chart of malignant cell subclusters in UMAP-defined group 1. **(H)** GO enrichment analysis bar chart of malignant cell subclusters in UMAP-defined group 2. **(I)** GO enrichment analysis bar chart of malignant cell subclusters in UMAP-defined group 3. **(J)** Heat map of the ITH expression program clustering. **(K)** Pathological region division of section GSM5494476. **(L)** Pathological region division of section PRJNA811533. **(M)** Box plot of MP expression in section GSM5494476. **(N)** Box plot of MP expression in section PRJNA811533.

Secondary unsupervised clustering was performed on all malignant cells to identify fine-grained subclusters (**Figure 1F**). To facilitate functional interpretation and avoid fragmented enrichment analysis of numerous individual subclusters, transcriptionally related subclusters located in adjacent neighborhoods of the UMAP embedding were consolidated into seven UMAP-defined groups. These groups represented transcriptional neighborhoods in the low-dimensional embedding. Functional enrichment identified distinct but partially overlapping biological themes across the seven UMAP-defined groups: epidermal differentiation and keratinization in group 1 (31–34); proliferation and MHC class II-related processes in group 2; proliferation and metabolic enzyme activity in group 3; stress responses in group 4; humoral immune regulation, tissue homeostasis, and transepithelial transport in group 5; glycosyltransferase-, serine-, and lipoprotein-related processes in group 6; and intermediate filament-associated features linked to EMT in group 7 (35) (**Figures 1G-I**; **Supplementary Figures S2A–D**). Together, these functional themes reflected multiple dimensions of malignant-cell heterogeneity, including differentiation, proliferation, metabolism, stress, immune-related activity, and EMT-associated features.

Previous studies have shown that ITH expression metaprograms (MPs) are the essential origin of ITH production (36). Therefore, to complement the cell-state resolution provided by the malignant-cell subclusters and UMAP-defined groups, we next examined transcriptional programs across individual patients. Unlike the cluster-based classifications above, meta-programs were derived by grouping co-expression programs identified independently within individual tumors and therefore captured transcriptional processes shared across patients and potentially across multiple malignant-cell subclusters. First, 87 ITH expression programs were identified across 17 OSCC patients, each defined by the 50 genes with the highest contribution scores (**Supplementary Table S2**). Hierarchical clustering based on Jaccard similarity grouped these programs into five MPs (**Figure 1J**): keratinocyte differentiation (MP3; KRT14, KRT17, and KRT5), epidermal cell differentiation (MP5; ANXA1, CSTA, KRT16, KRT6, S100A7, SFN, and SPRR1B), symbiotic interaction (37–39) (MP4; IFITM1 and IFITM3), fibroblast proliferation (40) (MP2; CD74, FTH1, and MIF), and stress response (41–43) (MP1; FOS and JUNB). MP3 and MP5 were functionally concordant with the keratinization- and differentiation-related features of UMAP-defined group 1, whereas the other MPs captured additional transcriptional features across patients.

To further clarify the role of MPs from a spatial perspective, we integrated scRNA-Seq and ST-Seq data to analyze the spatial expression patterns of the five MPs in primary OSCC tissues. Spots were first divided into three pathological regions (**Figures 1K, L**). All MPs showed significant expression heterogeneity across these regions, indicating distinct spatial preferences (**Figures 1M, N**). MP1, MP3, and MP5 were highly expressed in the tumor core (TC) region, consistent with their roles in defining the basic malignant traits of OSCC. Notably, among all MPs, MP2 demonstrated a distinctive, significantly high expression pattern in the tumor front (TF) region, which is typically characterized by active cell proliferation and invasive growth and serves as the critical leading edge for tumor-host interactions (9, 44).

Collectively, malignant-cell subclusters and meta-programs captured complementary dimensions of OSCC ITH, while the preferential enrichment of the fibroblast proliferation MP at the tumor front suggested a connection between malignant-cell heterogeneity and stromal-associated spatial features. This observation prompted us to further characterize CAF heterogeneity and its potential role in the OSCC microenvironment.

### PI3+ CAF have potential immunomodulatory effects

Cancer-associated fibroblasts (CAFs) are a specific subtype of fibroblast found in the tumor microenvironment. They have been proven to promote tumor progression, metastasis, angiogenesis, and immune escape through various mechanisms (45–49). Since fibroblast proliferation MP showed a distinct expression pattern in the TF region, we analyzed the role of CAFs in OSCC ecology. Cells initially assigned to the broad fibroblast compartment during major-cell-type annotation were extracted and independently reclustered into 15 subclusters (**Supplementary Figure S3A**). Subclusters predicted as other non-fibroblast cell were excluded. This refinement procedure yielded 3,487 CAFs for subsequent analysis. Representative differentially expressed genes of the retained CAF subclusters included COL1A1, COL3A1, COL6A3, CTHRC1, POSTN, and CFD (**Supplementary Figure S3B**).

All the CAFs mentioned above were divided into 7 subclusters through secondary unsupervised clustering, including 3 inflammatory CAFs (iCAF) and 4 myofibroblastic CAFs (myCAF) subclusters (**Figure 2A**). Each of the two CAF subtypes overexpressed their specific known marker genes, respectively (**Figure 2B**). Notably, the myofibroblastic CAF-PI3 (PI3+ CAF) subcluster not only overexpressed myofibroblast marker genes such as COL1A1 but also specifically overexpressed S100A8 and S100A9, which are closely related to myeloid cells and involved in immune regulation (50–52). In fact, myofibroblasts play a unique role in human immune regulation, such as promoting immune rejection and immunosuppression (53). Therefore, the PI3+ CAF subcluster may significantly influence the modeling of the OSCC immune microenvironment.

**Figure 2:**
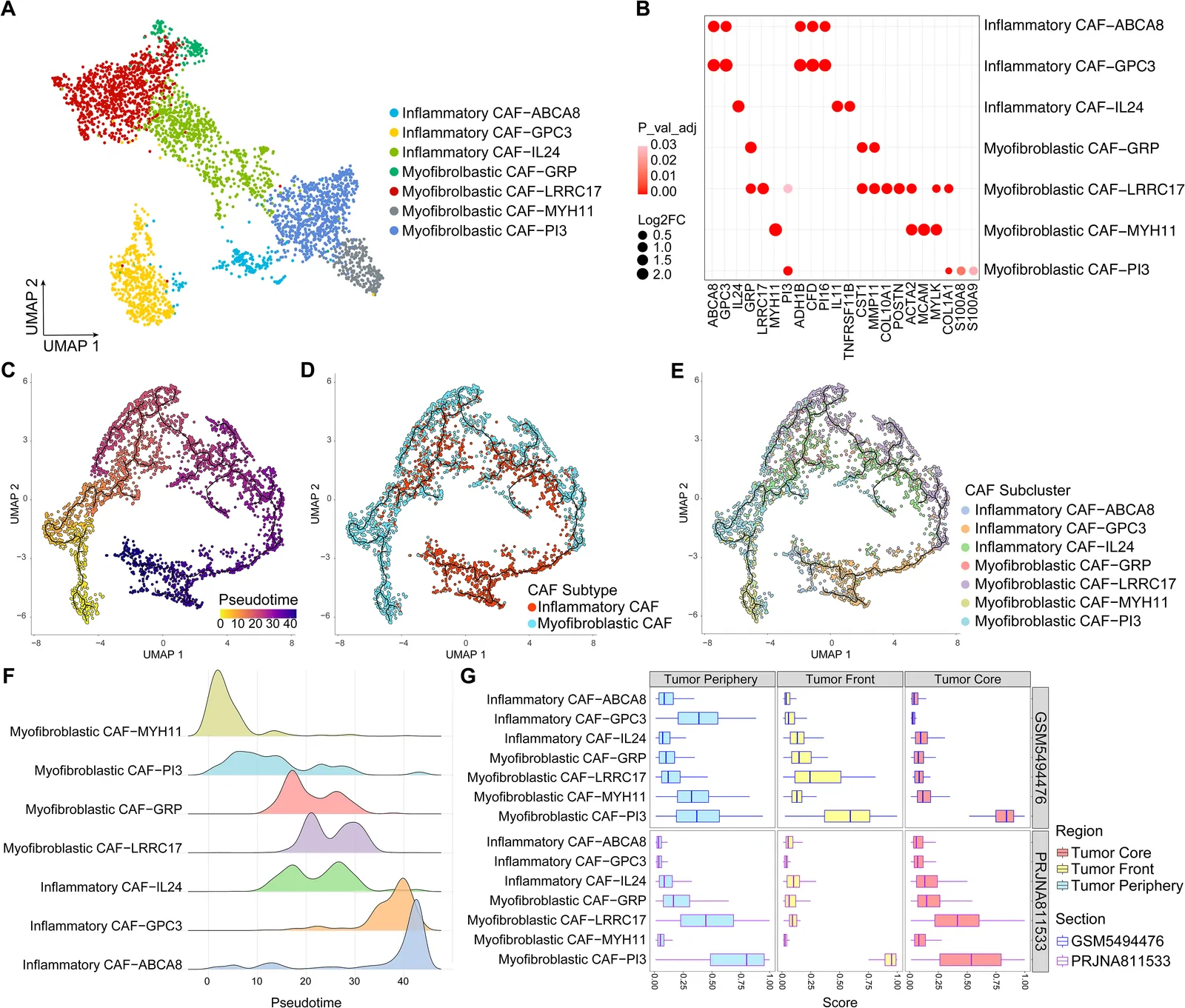
**(A)** UMAP dimensionality reduction mapping plot of CAF subclusters. **(B)** Bubble plot of differential gene expression of CAF subclusters. **(C)** Pseudotime trajectory mapping plot of the CAF pseudotime value. **(D)** Pseudotime trajectory mapping plot of CAF subtypes. **(E)** Pseudotime trajectory mapping plot of CAF subclusters. **(F)** Ridge plot of pseudotime value distribution of CAF subclusters. **(G)** Box plot of the spatial distribution of CAF subclusters.

Fibroblasts can create an inflammatory environment by increasing the secretion of proinflammatory cytokines and chemokines and may undergo phenotypic changes (54). Previous studies have demonstrated substantial plasticity between myofibroblastic and inflammatory CAF states, with TGF-β signaling favoring myCAF-associated programs and IL-1/JAK/STAT signaling promoting iCAF-associated programs (55). Based on this established biological framework, together with the enrichment of myCAF-associated markers at one end of the inferred trajectory, we designated the myCAF-enriched end as the root of the pseudotime analysis. The resulting ordering was consistent with a transition from myofibroblastic toward inflammatory CAF-associated states, although this represents a biologically informed computational interpretation rather than direct lineage tracing. In the middle portion of the trajectory, myCAFs and iCAFs coexisted, whereas iCAFs predominated toward the later pseudotime intervals (**Figures 2C–E**). The myCAF subclusters GRP and LRRC17 overlapped with the iCAF subcluster IL24 along the trajectory (**Figure 2F**), consistent with an intermediate region between the two transcriptional states. Notably, PI3+ CAF occupied an earlier myofibroblastic-associated position relative to iCAF-IL24 along the inferred continuum. Together with its spatial enrichment in the tumor core and tumor front regions (**Figure 2G**), these findings placed PI3+ CAF within a myofibroblastic-associated state preceding the emergence of stronger inflammatory CAF features. Given its expression of the myeloid-associated genes S100A8 and S100A9, we next examined the spatial and intercellular relationships between PI3+ CAF and myeloid cells.

### CAF–myeloid interactions in the OSCC microenvironment

To analyze the interaction between myeloid cells and CAFs, 9591 myeloid cells were clustered into 11 subclusters (**Supplementary Figure S4A**). Among these, subclusters 1 and 11 highly expressed marker genes of squamous epithelial cells (KRTDAP and S100A7) and immature B cells (CD79A, MS4A1, VPREB3, and FCER2), respectively (**Supplementary Figure S4B**). Consequently, these two subclusters were removed, and 7792 myeloid cells were retained for further analysis. All myeloid cells were then grouped into 8 detailed subclusters, comprising 5 subtypes (**Figures 3A, B**). Among all myeloid cells, the subcluster APC_Myeloid showed a notably high enrichment in the TC and TF pathological regions (**Figure 3C**, **Supplementary Figure S5A-B**). Interestingly, this spatial preference is nearly consistent with the previously mentioned PI3+ CAF subcluster.

**Figure 3:**
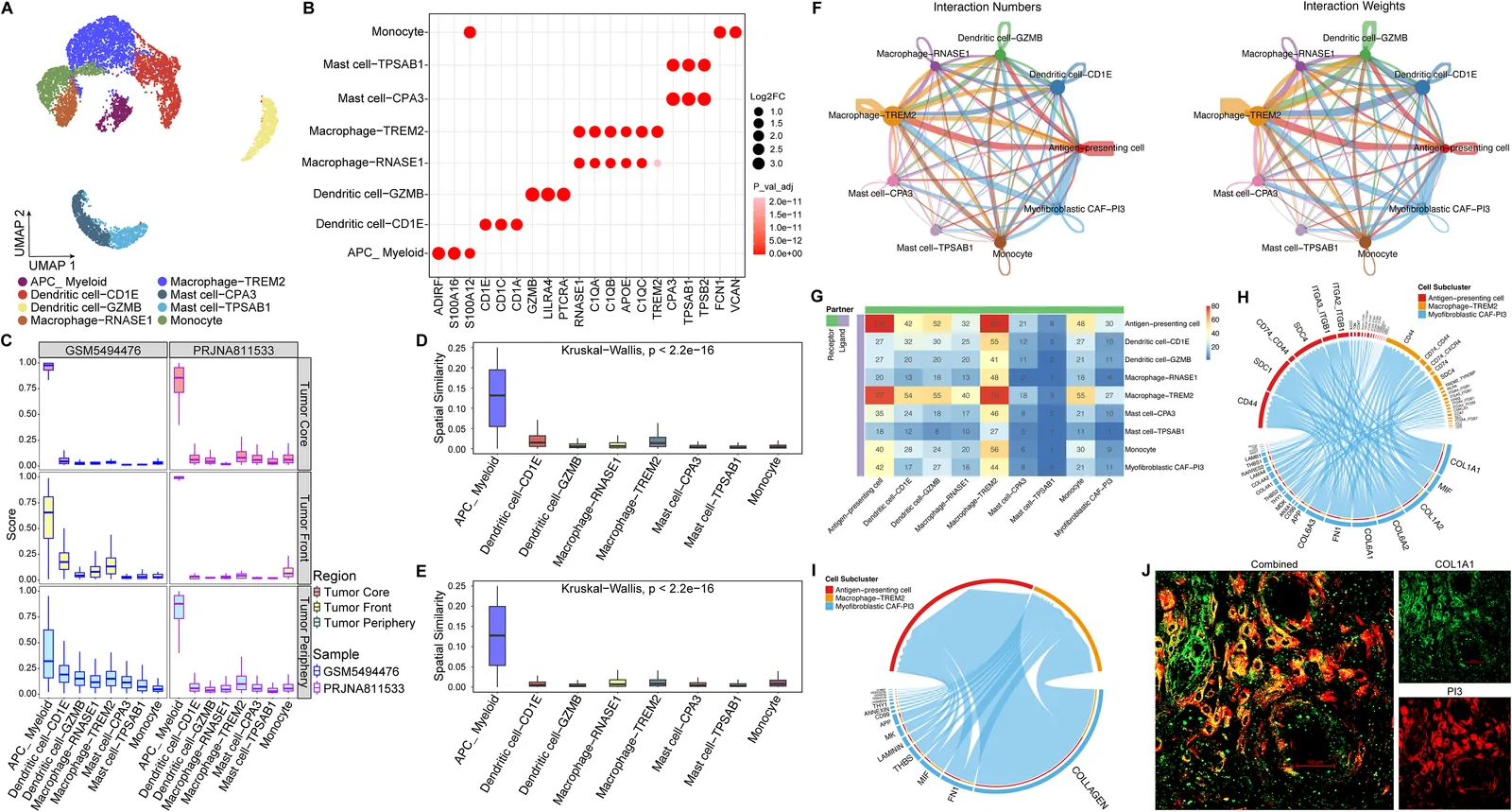
**(A)** UMAP dimensionality reduction mapping plot of myeloid cell subclusters. **(B)** Bubble plot of differential gene expression of myeloid cell subclusters. **(C)** Box plot of the spatial distribution of myeloid cell subclusters. **(D)** Box plot of spatial similarity between PI3+ CAF subcluster and myeloid cell subclusters in section GSM5494476. **(E)** Box plot of spatial similarity between PI3+ CAF subcluster and myeloid cell subclusters in section PRJNA811533. **(F)** Interaction network diagram from CellChat. **(G)** Interaction heat map from CellPhoneDB. **(H)** Chord diagram of interaction genes between PI3+ CAF subcluster and APC_Myeloid and TREM2 macrophage subclusters. **(I)** Chord diagram of interaction pathways between PI3+ CAF subcluster and APC_Myeloid and TREM2 macrophage subclusters. **(J)** Immunofluorescence staining slide images.

To quantify the spatial relationship between PI3+ CAF and the myeloid subclusters, we calculated spatial similarity in two independent spatial transcriptomic sections. This metric integrates the deconvolved abundances of two cell subclusters across spatial spots with the distances between those spots; higher values indicate that the two subclusters are more abundant in spatially proximal spots and therefore show stronger spatial colocalization. Among all myeloid subclusters, APC_Myeloid showed the highest spatial similarity with PI3+ CAF in both GSM5494476 and PRJNA811533 (**Figures 3D, E**). These results indicated a reproducible spatial association between PI3+ CAF and APC_Myeloid in OSCC tissues.

We next performed single-cell communication analysis to investigate the potential direction and molecular basis of this association. CellChat predicted PI3+ CAF as a major signal sender, whereas APC_Myeloid showed the strongest incoming interaction with PI3+ CAF among the myeloid subclusters, followed by macrophage-TREM2 (**Figure 3F**). An independent analysis using CellPhoneDB produced concordant results (**Figure 3G**), further supporting a directional communication axis from PI3+ CAF to APC_Myeloid.

To determine whether this communication axis was spatially organized within OSCC tissues, we further performed spatial ligand–receptor and directional cell–cell interaction analysis using stLearn. In both spatial transcriptomic datasets, the PI3+ CAF-to-APC_Myeloid interaction exhibited the highest aggregate significant cell–cell interaction count among all evaluated PI3+ CAF-to-myeloid interactions (**Supplementary Figure S6B, D**). In GSM5494476, 136 ligand–receptor pairs supported the PI3+ CAF-to-APC_Myeloid interaction, which showed a substantially higher total significant cell–cell interaction (CCI) count than the interactions involving the other myeloid receiver populations. In PRJNA811533, PI3+ CAF-to-APC_Myeloid again showed the highest total significant CCI count. Notably, although 106 ligand–receptor pairs were identified for the PI3+ CAF-to-monocyte interaction in PRJNA811533, compared with 38 pairs for PI3+ CAF-to-APC_Myeloid, its aggregate significant CCI count remained markedly lower. This indicated that the stronger APC_Myeloid interaction was not simply attributable to the number of ligand–receptor pairs included in the analysis.

We subsequently constructed cell-pair-specific composite spatial hotspot maps by integrating the significant ligand–receptor hotspot scores with the corresponding significant CCI counts for the PI3+ CAF-to-APC_Myeloid interaction. Spatially localized communication hotspots were detected in both OSCC sections (**Supplementary Figure S6A, C**). These hotspots were predominantly distributed within tumor-containing areas, including the tumor front and tumor core regions, indicating that the PI3+ CAF–APC_Myeloid communication axis was spatially organized within specific tumor niches rather than uniformly distributed throughout the tissue.

At the molecular level, PI3+ CAF predominantly expressed collagen-family ligands, including COL1A1, COL1A2, COL6A1, COL6A2, and COL6A3, whereas APC_Myeloid and macrophage-TREM2 expressed candidate downstream receptors such as CD44, CD74, and SDC4 (**Figure 3H**). Among the signaling pathways associated with these interactions, collagen signaling was the most prominent (**Figure 3I**), suggesting that collagen-family ligands represent a major component of PI3+ CAF-associated potential communication with downstream myeloid populations. Immunofluorescence staining of paraffin-embedded OSCC tissues further confirmed the co-expression of PI3 and COL1A1 (**Figure 3J** and **Supplementary Figure S7**), providing tissue-level support for the collagen-producing phenotype of PI3+ CAF.

Increased CAF activity and collagen-matrix deposition have been associated with the formation of physical barriers in the tumor microenvironment and resistance to immune-cell infiltration (56, 57). In addition, CD44 expressed by APC_Myeloid may interact with collagen-family ligands (58). Collectively, the spatial colocalization, independent single-cell communication analysis, spatial ligand–receptor and directional cell-pair interaction mapping, and immunofluorescence validation support a model in which PI3+ CAF participates in the organization of an immunosuppressive OSCC niche through collagen-associated signaling to APC_Myeloid. This interaction may alter the local extracellular matrix and interfere with the normal interaction between antigen-presenting myeloid cells and T cells, thereby contributing to impaired antitumor immune responses.

### The exhaustion evolution of CD8+ T cell

Currently, the immunotherapy used to treat OSCC mainly involves PD-1 inhibitors (5, 6). However, only a small percentage of OSCC patients respond to immunotherapy, and there is a lack of reliable biological markers to assess their sensitivity to such treatment (3). Part of this issue stems from the unclear immunosuppressive mechanisms within the OSCC microenvironment (59). Therefore, our study performed a multidimensional analysis of T cells, which are key players in the anti-tumor immune response. After secondary quality control, 31,682 T cells remained for further analysis. Unsupervised clustering identified 10 T cell subclusters derived from 7 T cell subtypes (**Figure 4A**), including CD4+ exhausted T cells (CD4_Exhau), CD4+ regulatory T cells (CD4_Treg), CD8+ cytotoxic T cells (CD8_Cyto), CD8+ memory T cells (CD8_Mem), CD8+ naive T cells (CD8_Naive), inflammatory NKT cells (NK_Inf), and proliferating T cells (T_Pro). Naive, memory, and cytotoxic CD8+ T cell subclusters were located close together on the UMAP, suggesting a developmental and differentiation pathway for CD8+ T cells. Each T cell subcluster highly expressed marker genes specific to its subtype, confirming the accuracy of the unsupervised clustering and annotation. For instance, inflammatory NKT cells expressed FCER1G and SH2D1B, whereas proliferating T cells expressed MKI67 and CDK1 (**Figure 4B**).

**Figure 4:**
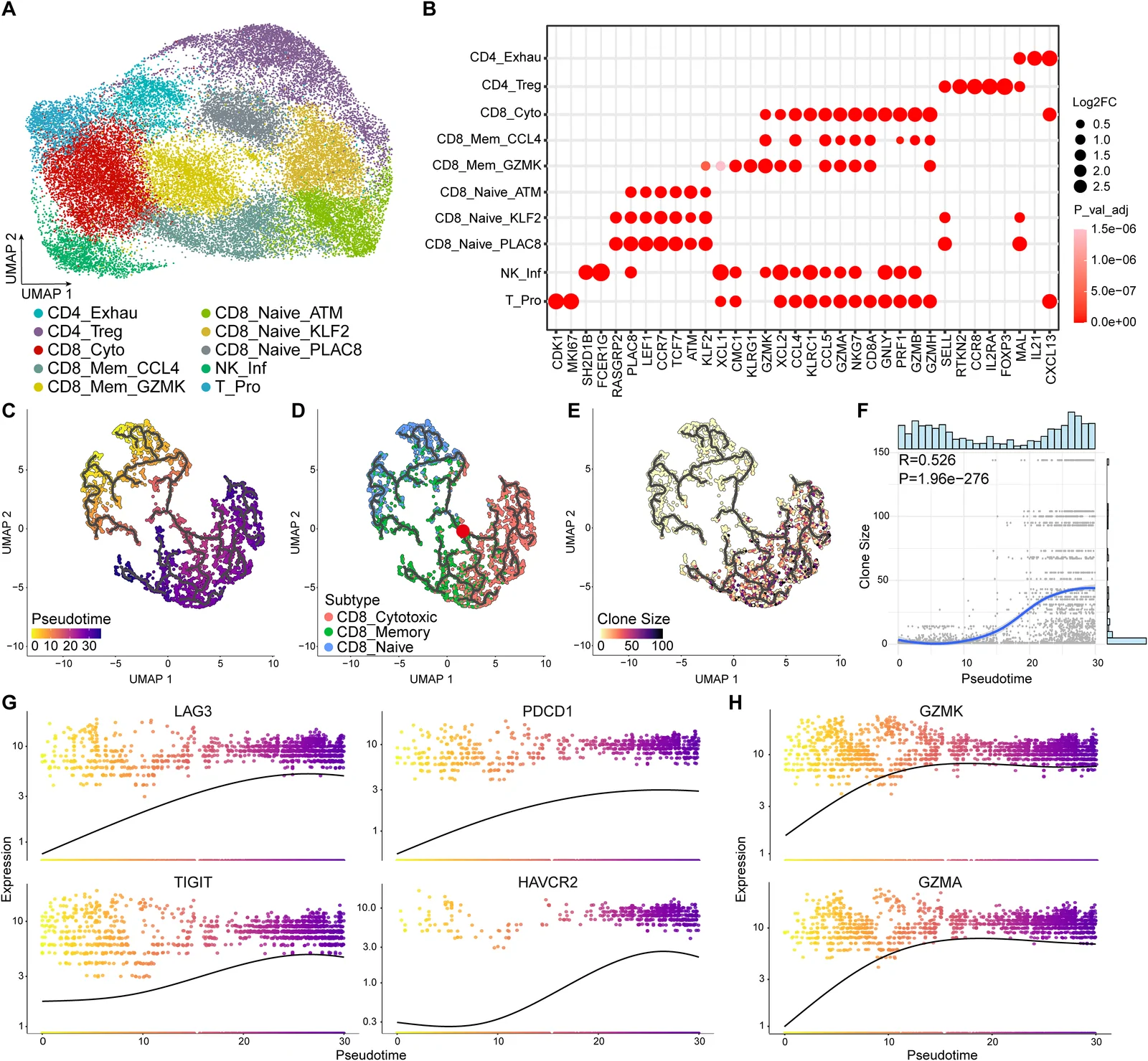
**(A)** UMAP dimensionality reduction mapping plot of T cell subclusters. **(B)** Bubble plot of differential gene expression of T cell subclusters. **(C)** Pseudotime trajectory mapping plot of CD8+ T cell pseudotime values. **(D)** Pseudotime trajectory mapping plot of CD8+ T cell subtypes. **(E)** Pseudotime trajectory mapping plot of CD8+ T cell clone size. **(F)** Correlation-fitting scatter plot between pseudotime values and clone size of CD8+ T cells. **(G)** Expression-trend-fitting scatter plot of exhaustion-phenotype marker gene expression versus pseudotime values for CD8+ T cells. **(H)** Expression trend fitting scatter plot between pre-dysfunction marker genes’ expression and pseudotime values of CD8+ T cells.

To examine CD8+ T-cell state dynamics together with TCR clonality, we next focused on the GSE212797 cohort, which provided paired scRNA-Seq and scTCR-Seq data. A total of 4,541 CD8+ T cells from four OSCC patients with paired transcriptomic and TCR information were retained for this analysis. Monocle3 was used to infer a pseudotemporal trajectory, along which naive, memory, and cytotoxic CD8+ T-cell states occupied distinct positions (**Figures 4C, D**). Previous studies have shown that antigen-stimulated naive CD8+ T cells can acquire effector/cytotoxic and memory-associated programs, whereas persistent antigen exposure can promote exhaustion-associated differentiation (60). Consistent with this established biological framework, the inferred trajectory began with naive-associated states and progressed toward cytotoxic states, with one branch also containing memory-associated cells. A distinct bifurcation point was observed midway through the trajectory (**Figure 4D**), leading to one branch predominantly composed of cytotoxic CD8+ T cells and another containing both cytotoxic and memory-associated cells. This ordering is compatible with heterogeneous CD8+ T-cell state transitions following antigen exposure, although pseudotime does not establish a definitive temporal lineage relationship.

In the early stage of T cell evolution (naive T cell), TCR clonal expansion is minimal. However, over time, especially after the branch point, TCR clonal expansion occurs rapidly and extensively (**Figure 4E**). Since T cell clonal expansion is usually triggered by antigen stimulation, these results also reveal the entire response of CD8+ T cells to tumor antigens. Furthermore, some CD8+ T cells at the end of the trajectory even show extreme clonal expansion (clonal size >100), potentially indicating an exhausted CD8+ T cell phenotype. The clonal size of CD8+ T cells gradually increases with pseudotime, exhibiting a significant positive correlation (**Figure 4F**). Notably, the clonal size of CD8+ T cells does not increase at a constant rate throughout the process but instead shows a sharp increase in the middle stage, confirming the rapid response following initial tumor antigen stimulation.

Four exhaustion-related marker genes (LAG3, PDCD1, TIGIT, and HAVCR2) all showed an upregulation trend during CD8+ T cell development (**Figure 4G**). Interestingly, the peak upregulation of these genes occurred mid-stage, coinciding with the period when CD8+ T cells undergo rapid clonal expansion. Two pre-dysfunction marker genes (GZMK and GZMA) exhibited similar upregulation patterns (**Figure 4H**). However, it is notable that the peak upregulation of pre-dysfunction marker genes occurred earlier than that of exhaustion-related marker genes and aligned with the peak upregulation period of exhaustion markers. Overall, these findings reveal the gene expression characteristics of CD8+ T cells following stimulation with OSCC antigens. Specifically, pre-dysfunction marker genes begin to activate at the early stage of antigen stimulation. As their expression increases, exhaustion-related marker genes are activated, with their expression rising alongside the rapid clonal expansion of CD8+ T cells. This indicates that CD8+ T cells gradually develop an exhaustion phenotype and lose their anti-tumor immune function.

Collectively, the inferred pseudotemporal trajectory was consistent with a progression from naive and memory-associated transcriptional states toward cytotoxic and exhaustion-associated states. This trajectory shows that CD8+ T cells gradually shift from a cytotoxic state to an exhausted phenotype, with significant clonal expansion in the later stages, indicating that anti-tumor immunity becomes severely impaired, which may ultimately lead to immune escape by OSCC malignant cells.

### Regulatory and inflammatory NK T cell associations with CD8+ T cell exhaustion

To project the inferred CD8+ T cell trajectory onto the spatial transcriptomic sections, CD8+ T cells were divided into four ordered pseudotime intervals, termed levels 1–4, corresponding to successive quartiles of the trajectory, which served as reference states for spatial deconvolution. Since T cell infiltration usually occurs within the tumor region, we concentrated on the distribution patterns and dynamic changes of these four exhaustion levels in the TC and TF regions. The estimated spatial abundances of the four pseudotime intervals differed between the TC and TF regions, although the magnitude of these differences varied across intervals and sections (**Figures 5A, B**). In both sections, level 2 showed a relative shift toward higher abundance in the TF region compared with the other pseudotime intervals. This pattern coincided with an early-to-mid portion of the inferred trajectory, during which clonal expansion and exhaustion-associated gene expression began to increase.

**Figure 5:**
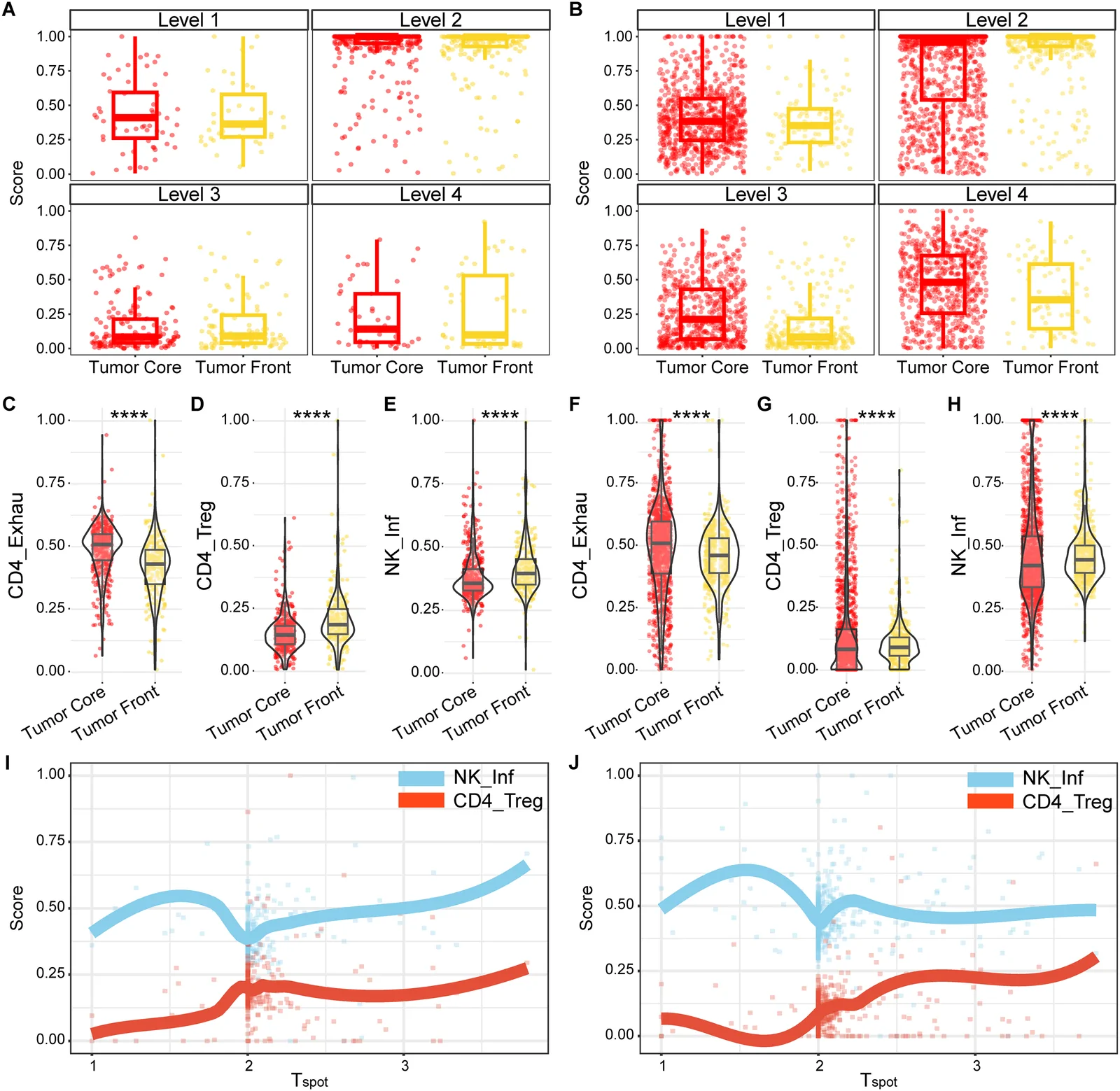
**(A)** Box plot of the spatial distribution of CD8+ T cells in section GSM5494476. **(B)** Box plot of the spatial distribution of CD8+ T cells in section PRJNA811533. **(C)** Combination plot of the spatial distribution of CD4+ exhausted T cells in section GSM5494476. **(D)** Combination plot of the spatial distribution of CD4+ regulatory T cells in section GSM5494476. **(E)** Combination plot of the spatial distribution of inflammatory NK T cells in section GSM5494476. **(F)** Combination plot of the spatial distribution of CD4+ exhausted T cells in section PRJNA811533. **(G)** Combination plot of the spatial distribution of CD4+ regulatory T cells in section PRJNA811533. **(H)** Combination plot of the spatial distribution of inflammatory NK T cells in section PRJNA811533. **(I)** Correlation fitting scatter plot between the infiltration level of CD4+ regulatory T cells and inflammatory NK T cells, and the continuous T_spot_ score in the tumor front region of section GSM5494476. **(J)** Correlation fitting scatter plot between the infiltration level of CD4+ regulatory T cells and inflammatory NK T cells, and the continuous T_spot_ score in the tumor front region of section PRJNA811533. **** P < 0.0001.

Previous studies have shown that regulatory T cells (Tregs) can inhibit the activation of cytotoxic T cells (61). In the tumor microenvironment, this inhibition can further promote the functional exhaustion of CD8+ T cells. Additionally, other T cell subtypes may exert co-inhibitory effects on CD8+ cytotoxic T cells (15). Therefore, to explore how other T cell subtypes influence the exhaustion process of CD8+ T cells, the spatial patterns of CD4+ exhausted T cells, CD4+ Tregs, and inflammatory NKT cells were first analyzed individually. All these T cell subtypes demonstrated significant distribution differences between the TC and TF regions (**Figures 5C–H**). Among them, CD4+ exhausted T cells exhibited higher infiltration in the TC region, whereas CD4+ Tregs and inflammatory NKT cells showed higher infiltration in the TF region. Since the level 2 CD8+ T cells also exhibited high infiltration characteristics in the TF region (**Figures 5A, B**), CD4+ Tregs and inflammatory NKT cells may be associated with the spatial emergence of exhaustion-related CD8+ T cell states. To examine how the abundance of other T cell subtypes varied along the inferred CD8+ T cell trajectory, we calculated a continuous spot-level trajectory score, T_spot_, as the weighted sum of the estimated proportions of levels 1–4, using weights of 1, 2, 3, and 4, respectively. Higher T_spot_ values therefore indicated a greater contribution from later pseudotime intervals and stronger exhaustion-associated characteristics, rather than assignment of a spot to a discrete exhaustion level. Because CD4+ Tregs and inflammatory NKT cells exhibited similar high levels of infiltration in the TF region, we then examined the relationships between T_spot_ and the estimated abundances of CD4+ Tregs and inflammatory NKT cells in TF spots. Most tumor-front spots had T_spot_ values clustered around 2 (**Figures 5I, J**). Around this interval of the continuous trajectory score, the estimated abundances of CD4+ Tregs and inflammatory NKT cells increased rapidly, indicating a swift infiltration process. After this period, the infiltration levels of both T cell subtypes gradually stabilized, showing a slow overall increase.

The spatiotemporal analysis above reveals a close connection between CD8+ T cells and both CD4+ regulatory T cells (Tregs) and inflammatory NKT cells. Specifically, during the phases of rapid clonal expansion and activation of the exhaustion phenotype in CD8+ T cells, CD4+ Tregs and inflammatory NKT cells showed significant co-infiltration with CD8+ T cells in the tumor front (TF) region of OSCC. This spatial and pseudotemporal concordance is consistent with a potential involvement of CD4+ Tregs and inflammatory NKT cells in immunosuppressive niches associated with CD8+ T cell exhaustion. The immunosuppressive role of CD4+ Tregs has been well documented in previous studies, and strategies aimed at depleting Tregs or altering their function (e.g., CAR-Tregs) show promise for boosting anti-tumor immunity and aiding tumor clearance (62–64). However, the immunosuppressive mechanisms of inflammatory NKT cells remain poorly understood. Terabe et al. (65) summarized the dual role of inflammatory NKT cells in tumor formation, proposing the existence of at least two different subtypes: one that enhances anti-tumor responses, and another that suppresses them. Therefore, inflammatory NKT cells are a promising new target for future immunotherapy in OSCC.

### Uncover OSCC ecotypes and coordinated cell programs based on the OSCC cell atlas

To complete the cellular reference atlas used for ecosystem-level deconvolution, we further characterized 8,602 B-lineage cells into eight subclusters representing six major states, including germinal-center B cells, immature B cells, naive B cells, plasma cells, precursor B cells, and professional antigen-presenting B cells (APC_B) (**Figures 6A, B**). Together with the previously defined malignant, T-cell, myeloid, and CAF subclusters, these B-cell states completed the reference used for subsequent bulk RNA-seq deconvolution and ecotype construction. Overall, the integrated atlas comprised 88,442 cells from 41 samples and resolved five major cell types, 22 cell subtypes, and 63 cell subclusters (**Figure 6C**).

**Figure 6:**
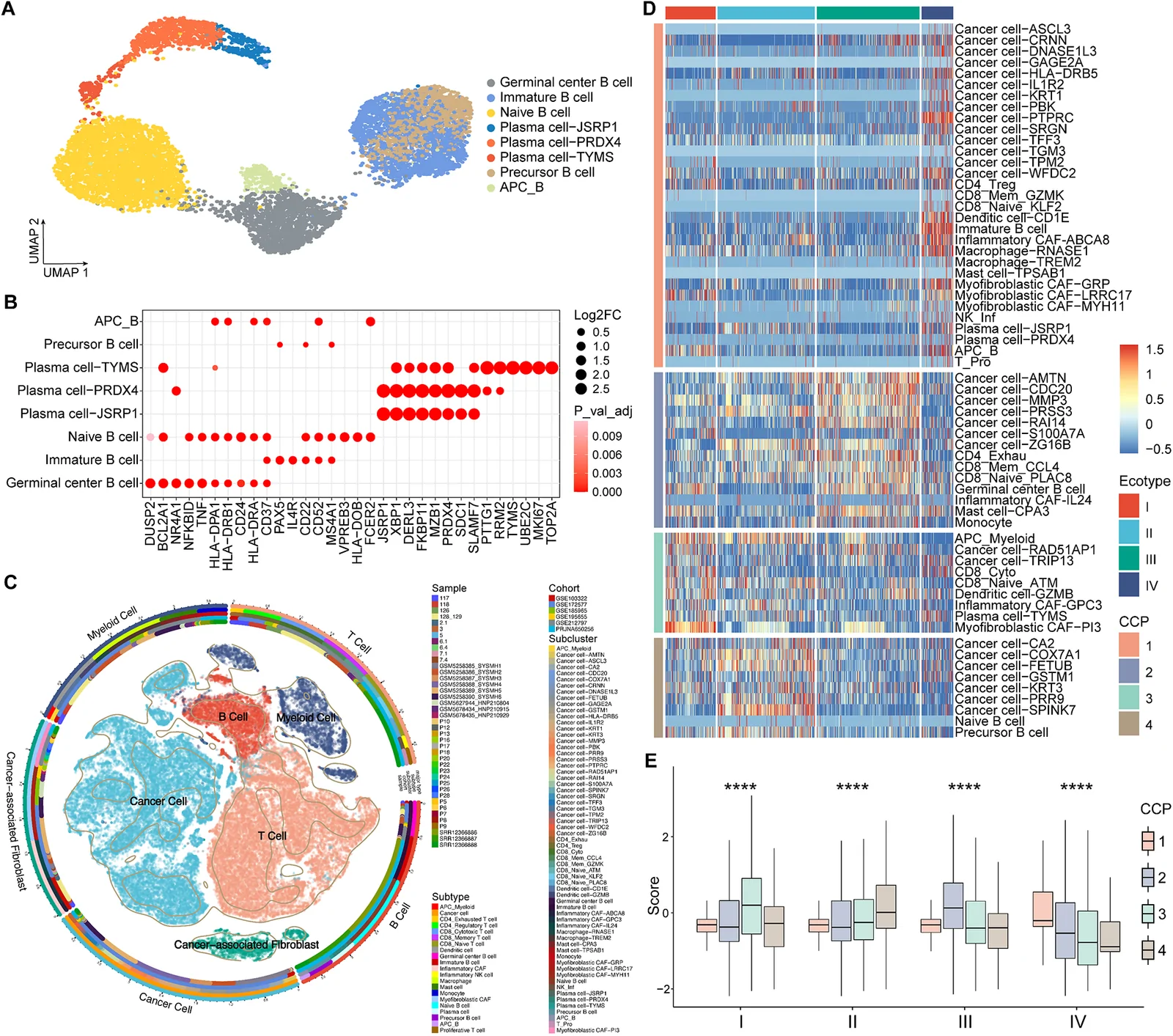
**(A)** UMAP dimensionality reduction mapping plot of B cell subclusters. **(B)** Bubble plot of differential gene expression of B cell subclusters. **(C)** Schematic diagram of the OSCC cell atlas. **(D)** Heat map of NMF clustering of OSCC ecotypes and CCPs. **(E)** Box plot of enrichment level of CCPs in each OSCC ecotype. **** P < 0.0001.

To extend the single-cell atlas to patient-level ecosystem organization, CIBERSORT was used to deconvolve bulk RNA-seq profiles from 1,017 patients across 18 cohorts using signatures derived from the 63 single-cell-defined subclusters, generating a patient-by-cell-subcluster composition matrix (**Supplementary Figure S8**). Nonnegative matrix factorization (NMF) column clustering grouped patients with similar inferred cellular compositions into four OSCC ecotypes, whereas NMF row clustering, following the framework of Chen et al. (66), grouped cell subclusters with coordinated abundance patterns across patients into four coordinated cell programs (CCPs) (**Figure 6D**). Thus, ecotypes represent patient-level ecosystem classes, whereas CCPs represent cross-patient co-abundance modules of already defined cell subclusters. Unlike malignant-cell meta-programs, which capture recurrent gene-expression programs within malignant cells, CCPs capture coordinated abundance patterns across multiple cellular populations. These frameworks therefore describe complementary organizational levels of OSCC heterogeneity.

Patients within the same ecotype showed a highly consistent cell subcluster makeup. For example, patients with ecotype I consistently showed high levels of the PI3+ CAF subcluster. Cell subclusters within the same CCP also demonstrated clear co-enrichment patterns. Specifically, dendritic cell-CD1E, immature B cells, inflammatory CAF-ABCA8, and macrophage-RNASE1 all consistently had higher enrichment in ecotype IV patients. CCP3, CCP4, CCP2, and CCP1 were preferentially enriched in ecotypes I, II, III, and IV, respectively (**Figure 6E**), linking patient-level ecotypes to cellular programs and providing a basis for subsequent molecular and clinical characterization.

### Biological profile of OSCC ecotypes

To compare the ecosystem-based ecotypes with traditional bulk-defined molecular subtypes and other clinical phenotypes, we analyzed data from 1,017 OSCC patients across 11 parameters: molecular subtype, prognosis, recurrence, HPV infection status, smoking history, drinking history, response to anti-PD-1 immunotherapy, age, sex, tumor grade, and clinical stage. Regarding traditional molecular subtypes (**Figure 7A**), ecotype I was primarily characterized by the mesenchymal subtype, whereas ecotype II was predominantly defined by the basal subtype. Ecotype III was also dominated by the mesenchymal subtype; however, unlike ecotype I, these patients showed a significantly higher proportion of the basal subtype. Ecotype IV was primarily associated with the atypical subtype. Survival distributions differed among the four OSCC ecotypes in the available cohorts (**Figure 7B**), with ecotype II showing a comparatively favorable survival pattern and ecotype III showing a less favorable pattern.

**Figure 7:**
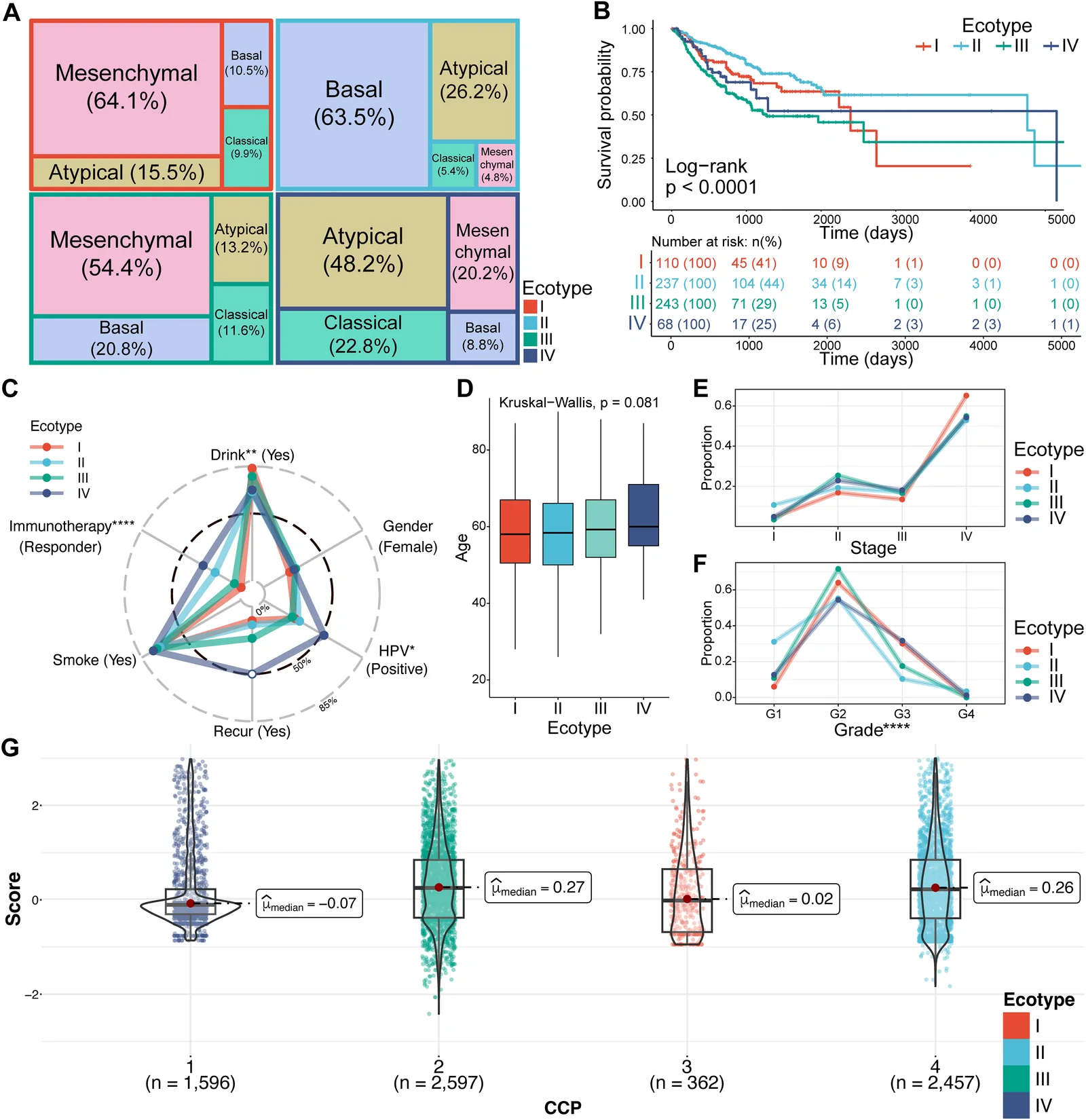
**(A)** Treemap of the relationship between OSCC ecotypes and traditional molecular subtypes. **(B)** Stratified KM survival curve based on OSCC ecotypes. **(C)** Radar plot of binary clinical features of OSCC ecotypes. **(D)** Box plot of the age distribution of each OSCC ecotype. **(E)** Line chart of the clinical-stage proportion for each OSCC ecotype. **(F)** Line chart of tumor grade proportion of each OSCC ecotype. **(G)** Enrichment level of malignant cell subclusters of CCPs in the corresponding highly enriched OSCC ecotype.

Among all binary clinical features, alcohol history, HPV infection status, and predicted response to anti-PD-1 immunotherapy showed significant differences across each ecotype (**Figure 7C**). Patients with ecotype IV were more likely to benefit from immunotherapy, while those with ecotype I showed almost no response, highlighting the strong potential of the OSCC ecotyping system for predicting immunotherapy outcomes. Furthermore, the proportion of HPV-positive patients in ecotype IV reached 50%, significantly higher than in other ecotypes. Since HPV infection is a known risk factor for OSCC (67–69), the strong association between OSCC ecotypes and HPV infection status further supports the effectiveness of our OSCC ecotyping system. It is also worth noting that although ecotype IV patients (only two) had the highest recurrence rate at 50%, this result may be due to chance. Additionally, ecotype III patients showed a relatively high recurrence rate, though it did not reach statistical significance, possibly due to the limited number of samples with recurrence data.

No significant differences in age were observed among patients across OSCC ecotypes (**Figure 7D**). Most OSCC patients were classified as stage IV (**Figure 7E**), which aligns with clinical reality, as OSCC is often diagnosed at advanced stages. Notably, the proportion of ecotype I patients in stage IV was the highest, suggesting that ecotype I patients may be more advanced. Conversely, ecotype II patients had the highest proportion in stage I, suggesting a possible association with early-stage disease. Significant differences in tumor grade were observed among patient ecotypes (**Figure 7F**). Specifically, ecotype II patients showed the highest proportion of grade 1 (G1), consistent with early-stage characteristics and was associated with a comparatively favorable survival pattern. To relate these clinical patterns to ecosystem composition, we next examined each ecotype in the context of its preferentially enriched CCP.

Ecotype I was characterized by enrichment of CCP3, including PI3+ CAF, APC_Myeloid, and CD8+ cytotoxic T cell subclusters, and showed a lower predicted response to immunotherapy in the available cohort. Together with the inferred PI3+ CAF–APC_Myeloid communication axis, this pattern raises the hypothesis that stromal–myeloid interactions may contribute to an immunosuppressive microenvironment despite the presence of cytotoxic T cell populations. However, the observed association does not establish that PI3+ CAF is the cause of immunotherapy resistance, and the potential therapeutic value of targeting this subcluster requires functional and clinical validation. Regarding traditional molecular subtypes, these patients primarily belong to the mesenchymal subtype. Chung et al. revealed that HNSCC patients of the mesenchymal type usually show high expression of fibroblast-related genes, including collagen subunit genes (7). This finding aligns with the high enrichment of the PI3+ CAF subcluster observed in ecotype I patients.

Ecotype II patients show a higher enrichment of CCP4 and proportion of early-stage cases in the available cohort. CCP4 is mainly composed of subclusters of malignant cells, indicating that other cell subclusters do not significantly influence the tumor microenvironment. As a result, its ecosystem structure is relatively simple, which aligns with the early clinical phenotype observed in these patients. Conversely, although CCP4 contains malignant cells, these patients have the best prognosis and lower recurrence rates, highlighting the important role of other cell subtypes in the OSCC ecosystem in tumor progression.

Ecotype III patients were highly enriched for CCP2 and had the poorest prognosis, with the highest recurrence rate. Their resistance to immunotherapy was also second only to that of ecotype I patients. Although CCP2 consists of three T cell subclusters—exhausted CD4+, memory CD8+, and naive CD8+ T cells—none of these subclusters can directly perform cytotoxic functions. Moreover, the presence of exhausted CD4+ T cells suggests an immunosuppressive environment. As a result, the low efficacy of immunotherapy may be due to a lack of effective infiltration by anti-tumor immune cells. Additionally, while malignant cell subclusters are found in all CCPs and show specific enrichment within their respective ecotypes, the absolute levels of these malignant subclusters vary. For example, although malignant subclusters such as ASCL3, GAGE2A, and TGM3 from CCP1 and are relatively enriched in ecotype IV patients, their absolute enrichment levels remain low (**Figure 6D**). In contrast, malignant cell subclusters within CCP2 display high absolute enrichment levels in these ecotype III patients (**Figure 7G**), consistent with a potentially more aggressive transcriptional profile. Overall, the combined effect of an immunosuppressive environment and a strongly aggressive phenotype likely leads to the poorest prognosis and highest recurrence in these patients.

Ecotype IV patients were highly enriched in CCP1, which is characterized by a strong association with HPV infection and the highest smoking prevalence. CCP1 includes numerous immune cell subclusters, such as five T cell, four myeloid, and four B cell subclusters. As a result, this ecotype exhibits high immune infiltration, a trait closely linked to HPV infection (70, 71). Additionally, these patients demonstrated the greatest sensitivity to immunotherapy. This may be due to HPV-positive tumors generally exhibiting increased immune infiltration, leading to a more robust anti-tumor immune response (72, 73). In terms of traditional molecular subtypes, these patients primarily belong to the atypical and classical groups. Walter et al. reported that the atypical subtype is notably associated with HPV positivity, while the classical subtype is linked to a history of heavy smoking (8). These findings reflect the clinical features observed in patients with ecotype IV.

Across the four ecotypes, ecotype-specific cellular programs showed associations with molecular subtypes, survival patterns, and selected clinical characteristics. However, these findings should be interpreted as exploratory trends because of unequal group sizes, incomplete clinical annotation, and potential confounding by HPV status and other clinical variables.

### Exploratory prediction of ecotype-associated drug sensitivity

We next examined whether these ecosystem-level differences were also reflected in predicted sensitivity to standard chemotherapy and candidate compounds. These in silico predictions were generated using oncoPredict models trained on OSCC cell-line gene expression and drug-response datasets and applied to patient bulk transcriptomic profiles. Currently, three first-line chemotherapy regimens are used for the treatment of OSCC: Docetaxel + Platinum + Fluorouracil (TPF), Docetaxel + Platinum (TP), and Platinum + Fluorouracil (PF). However, non-specific cytotoxicity increases the risk of adverse events (74, 75). Therefore, we explored whether the OSCC ecotypes were associated with differential predicted sensitivity to standard chemotherapy agents. Overall, drug sensitivity to carboplatin, fluorouracil, and docetaxel varied significantly (p< 0.0001) across the four OSCC ecotypes. Specifically, carboplatin showed high sensitivity in ecotype II patients but high resistance in ecotype I (**Supplementary Figure S9A**). Fluorouracil was highly effective in ecotype II patients but resistant in ecotype I and ecotype IV (**Supplementary Figure S9B**). Docetaxel was highly effective in ecotype IV patients but resistant in ecotype I (**Supplementary Figure S9C**). In summary, ecotype I patients showed resistance to all the drugs mentioned. Ecotype II patients demonstrated high sensitivity to both carboplatin and fluorouracil, indicating they might be better suited for the PF regimen. Patients with ecotype III had average sensitivity levels to these drugs. Ecotype IV patients showed high sensitivity to docetaxel but resistance to fluorouracil, suggesting they may respond better to the TP regimen.

Given the high resistance of ecotype I and ecotype III patients to traditional first-line chemotherapy agents, we further explored candidate drugs suitable for these two ecotypes. AUY, dasatinib, GSK1059615, and obatoclax were identified as candidate drugs for patients with ecotype I (**Supplementary Figures S9D-G**). Notably, although these drugs demonstrated specific sensitivity within the ecotype I patients, they also exhibited high sensitivity across the entire OSCC cohort. For instance, GSK1059615 displayed similar sensitivity across ecotype I, II, and III patients, with a median drug-sensitivity area under the curve (AUC) below 0.5. Consequently, these candidate drugs are not only applicable to ecotype I patients but may also possess broad-spectrum therapeutic potential for OSCC. Current studies have demonstrated that in the context of human oral cancer, dasatinib can mediate potent anti-tumor growth, anti-angiogenic, and pro-apoptotic effects by modulating multiple targets (e.g., Src and EGFR) (76). Additionally, both GSK1059615 and obatoclax have been shown to reduce proliferation in OSCC cell lines (77).

AR-42, AR-12, and belinostat were identified as candidate drugs for patients with ecotype III (**Supplementary Figures S9H-J**). Since the pathogenesis of HNSCC is closely linked to histone acetylation, recent studies have highlighted the significant therapeutic potential of belinostat, a histone deacetylase inhibitor, for HNSCC (74). Although AR-42 and AR-12 remain in the investigational stage, existing studies have shown that AR-42 can effectively inhibit the viability and proliferation of OSCC cells (78). Additionally, experimental evidence suggests that HNSCC cells are highly sensitive to AR-12 compared with normal mucosal cells (79).

Taken together, these analyses identified candidate compounds with differential predicted sensitivity across the OSCC ecotypes. Because the predictions were derived from cell-line pharmacogenomic resources, they should be interpreted as exploratory hypotheses for subsequent experimental validation rather than evidence of clinical efficacy. Additionally, because of the strong biological interpretability of our OSCC ecotyping system, this approach enables a deeper understanding of drug action mechanisms and promotes the development of new therapeutic targets.

### Clinical prediction models based on OSCC ecology

Our analysis showed that patients with different OSCC ecotypes have significant variations in several clinical features, including prognosis and response to immunotherapy. Since an OSCC ecotype reflects the overall cellular composition of tumor tissue, we further explored whether subcluster composition could predict individual clinical phenotypes. Unlike traditional gene-based prediction models, our models based on cellular composition can directly demonstrate how cell subclusters influence specific clinical outcomes, such as prognosis, through coefficients. This not only enhances understanding of how particular subclusters function during the initiation and progression of OSCC but also clearly shows the connection between the OSCC ecosystem and clinical phenotypes. Considering the current clinical situation of OSCC, we used the cell subcluster profiles of 1,017 OSCC patients to develop three prediction models for prognostic risk, recurrence risk, and immunotherapy sensitivity.

The prognostic risk prediction model includes 12 cell subclusters (**Supplementary Table S3**). Notably, the CPA3+ mast cell subcluster showed the highest coefficient, making it the most significant risk factor. A previous report has clarified the malignant, pro-tumorigenic roles of CPA3 in OSCC, and its expression has been linked to poor outcomes (80). In both the training cohort (**Figure 8A**) and external validation cohorts (**Figures 8B–D**), significant survival differences were observed between high- and low-risk groups stratified by this model. The high-risk group consistently showed worse survival in the evaluated cohorts, indicating that the model score was associated with prognosis across these datasets.

**Figure 8:**
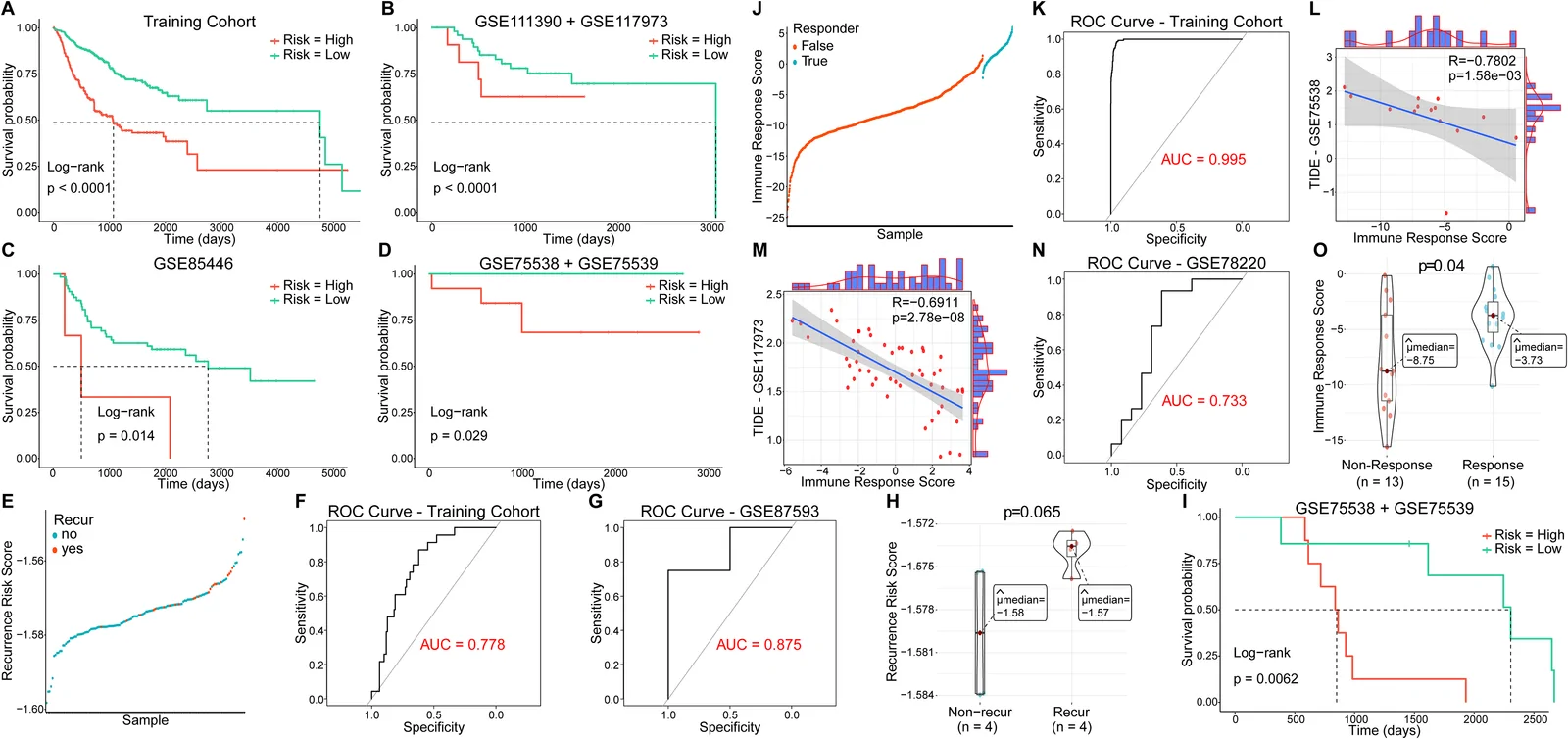
**(A)** KM curve of the model groups on the training cohort. **(B)** KM curve of the model groups on the combined data cohort of GSE111390 and GSE117973. **(C)** KM curve of the model groups on GSE85446. **(D)** KM curve of the model groups on the combined data cohort of GSE75538 and GSE75539. **(E)** Scatter plot of the correlation between model score and recurrence label. **(F)** ROC curve of the model on the training cohort. **(G)** ROC curve of the model on GSE87593. **(H)** Box plot of model score on GSE87593. **(I)** DFS survival curve of the model groups on the combined data cohort of GSE75538 and GSE75539. **(J)** Scatter plot of the correlation between model score and immunotherapy response label. **(K)** ROC curve of the model on the training cohort. **(L)** Scatter plot of the correlation between model score and TIDE score on GSE75538. **(M)** Scatter plot of the correlation between model score and TIDE score on GSE117973. **(N)** ROC curve of the model on GSE78220. **(O)** Box plot of model score on GSE78220.

The recurrence risk prediction model includes 56 cell subclusters (**Supplementary Table S4**). Of these, 33 subclusters were protective factors, while the remaining 23 were risk factors. Among all protective factors, the proliferative T cell subcluster (T_Pro) ranked highest. The most significant risk factor was the CD4+ regulatory T cell subcluster (CD4_Treg), followed closely by the TPM2+ malignant cell subcluster (Cancer cell-TPM2). CD4_Treg cells are a key immunosuppressive element within the tumor immune microenvironment (62–64). Our previous analysis showed that in the tumor front (TF) regions of OSCC, this subcluster promotes the transition of CD8+ T cells toward exhaustion, impairing anti-tumor immune responses. Therefore, an immunosuppressive environment may be a primary factor in local recurrence of OSCC. Additionally, TPM family members are mainly involved in tumor invasion and migration (81), which aligns with the finding that the Cancer cell-TPM2 subcluster is a major risk factor for OSCC recurrence. In the training cohort, 134 patient samples were scored using this model. Patients with higher scores were more likely to experience recurrence (**Figure 8E**), and the model achieved an AUC of 0.778 (**Figure 8F**). In the external validation cohort GSE87593, the model achieved a binary prediction AUC of 0.875 (**Figure 8G**). Moreover, scores in the recurrence group were significantly higher than those in the non-recurrence group (**Figure 8H**). These results indicate that the recurrence-risk score was associated with recurrence status in the evaluated datasets. In the combined cohort of GSE75538 and GSE75539, there were significant differences in disease-free survival (DFS) between high- and low-recurrence risk groups based on model scores (**Figure 8I**). Notably, this divergence became more evident over the long term (around two years). Overall, the recurrence-risk score showed associations with recurrence status and DFS in the evaluated cohorts; however, the small external validation cohorts preclude firm conclusions regarding predictive accuracy or robustness.

The immunotherapy sensitivity prediction model includes 36 cell subclusters (**Supplementary Table S5**). Of these, 17 subclusters have negative coefficients, indicating risk factors that limit patients’ benefit from immunotherapy. The remaining 19 subclusters have positive coefficients, indicating protective factors that promote therapeutic benefit from immunotherapy. Notably, among all risk factors, the top three were myofibroblastic CAF-PI3, naive B cells, and mast cell-CPA3. The negative coefficient of the PI3+ CAF subcluster was consistent with its immunosuppressive characteristics identified in the preceding analysis, although the model coefficient does not independently validate a causal role in immunotherapy resistance. Among all protective factors, proliferative T cells ranked first with a coefficient of 82.37, substantially higher than the second-ranked coefficient of 33.29. Interestingly, this subcluster was also identified as the primary protective factor in the recurrence risk prediction model. Consequently, the positive coefficients assigned to proliferative T cells in both models were consistent with greater tumor immune dysfunction and exclusion (TIDE)-inferred immunotherapy sensitivity and lower estimated recurrence risk. This is likely because the rapid proliferation of T cells provides strong support for systemic anti-tumor immune functions. Meanwhile, the CD8+ cytotoxic T cell, which serves as the direct effector of anti-tumor cytotoxicity, also emerged as a protective factor in this model. In the development cohort, model scores differed between the TIDE-inferred response and non-response groups (**Figure 8J**), with an apparent AUC of 0.995 (**Figure 8K**). In five external validation cohorts, model scores consistently showed a negative correlation with TIDE scores (**Figures 8L, M**, **Supplementary Figure S10**). On the other hand, the model also achieved an AUC of 0.733 in the melanoma cohort (**Figure 8N**), with patients responding to immunotherapy exhibiting significantly higher scores than the non-responder group (**Figure 8O**). In summary, the model score was associated with TIDE-inferred immunotherapy response in the development cohort, TIDE scores in the external OSCC cohorts, and observed immunotherapy response in the melanoma cohort. However, OSCC validation was primarily based on a computational surrogate, whereas the cohort with observed treatment responses was derived from melanoma. These findings therefore provide preliminary evidence of model discrimination.

Overall, the three cell-composition-based models showed associations with prognosis, recurrence, and TIDE-inferred immunotherapy sensitivity across the evaluated datasets. Penalization and 10-fold cross-validation were used to constrain model complexity; however, these procedures do not eliminate overfitting, and performance measured in the development cohorts represents apparent performance. Because the models were constructed from deconvolved bulk profiles and some validation cohorts were small or indirect, they should be regarded as exploratory and require prospective validation in independent OSCC cohorts with well-annotated clinical outcomes.

## Methods

### Data source and preprocessing

scRNA-Seq data were obtained from five cohorts (accession ID: GSE103322 (30), GSE172577 (82), GSE212797 (83), GSE195655 (84), and GSE185965 (85)) from the Gene Expression Omnibus (GEO) database (86) and one cohort (accession ID: PRJNA650256 (87)) from the Sequence Read Archive (SRA) database (88). Only primary OSCC samples were retained. Details of each scRNA-Seq data cohort are shown in **Supplementary Table S6**. For upstream FastQ data, transcriptome alignment and quantification were performed using the Cell Ranger software (Version 6.1.2) with default parameters. The refdata-gex-GRCh38-2020-A was the reference genome profile. Then, the gene expression profiles of the six cohorts above were integrated, yielding a total of 117,245 cells from 41 samples and 18,167 intersection genes. Cells with fewer than 200 expressed genes or mitochondrial genes exceeding 20% are usually low-quality cells, while cells with more than 6,000 expressed genes are usually doublets (87). Therefore, only retained 114,148 cells with expressed genes greater than 200 and less than 6,000, and mitochondrial genes less than 20% for downstream analysis.

To parse the spatial features of OSCC, ST-Seq data of OSCC tumor tissues were obtained from GEO database (accession ID: GSE181300 (89)) and SRA database (accession ID: PRJNA811533 (90)), respectively. Details of each ST-Seq data cohort are shown in **Supplementary Table S7**. The 10X Visium ST-Seq data from the SRA database were processed by Space Ranger software (Version 2.0.1) based on the default parameters. The chemistry and slide serial number were set according to the source article (90).

To construct the dynamic CD8+ T cell exhaustion evolutionary trajectory, scTCR-Seq data were collected from the GEO database (accession ID: GSE212797 (83)). This data cohort contained 7633 cells from 4 OSCC patients, along with the corresponding TCR clonotype information. Based on the cell annotation labels provided in the source article (83), only 4541 CD8+ T cells with paired scRNA-Seq data and scTCR-Seq data were retained for subsequent analysis.

To robustly identify ecotypes in the OSCC patient population, our study integrated bulk RNA-Seq data and clinical information of 1017 primary OSCC patients from 18 data cohorts. Of these, 17 data cohorts were obtained from the GEO database (accession ID: GSE202048 (91), GSE208576 (92), GSE213862 (93), GSE217142 (94), GSE222673 (95), GSE246050 (96), GSE256047 (97), GSE31056 (98), GSE41613 (99), GSE42743 (100), and GSE65858 (101)), and another data cohort was obtained from the The Cancer Genome Atlas (TCGA) database (Project ID: TCGA-HNSCC (102)). Only samples with a sampling site in the oral cavity were retained. A comprehensive compendium of sample data from each cohort is delineated in **Supplementary Table S8**. The gene expression profiles from the 18 data cohorts above were integrated using 12,530 intersection genes. The batch effect was corrected using the function “Combat” in the R package sva (103).

To investigate whether patients from each OSCC ecotype are differentially sensitive to current chemotherapeutic regimens, we collected gene expression profiles and drug sensitivity data of cell lines from the Cancer Cell Line Encyclopedia (CCLE) database (104) (including Genomics of Drug Sensitivity in Cancer (GDSC) V1 and V2 (105), Cancer Therapeutics Response Portal (CTRP) V2 (106), and Profiling Relative Inhibition Simultaneously in Mixtures (PRISM) (107)). Only OSCC cell lines were retained for subsequent analysis.

### Dimension reduction and unsupervised clustering

The R package Seurat (version 4) (21) was used to perform single-cell unsupervised clustering analysis. The function “FindVariableFeatures” was first used to identify highly variable genes (HVGs) that effectively reflect biological differences between cells. Subsequently, the gene expression profile was normalized using the function “ScaleData”, and principal component analysis (PCA) was performed using the function “RunPCA” to reduce dimensionality. The steps for selecting the best principal components (PCs) are as follows: 1. Perform JackStraw (108) analysis and select PCs with p<0.05. 2. Use the function “ElbowPlot” to select PCs that appear near the inflection point of the line chart. 3. Use the above candidate PCs for unsupervised clustering and manually check the results to determine the best PCs. Since our study integrated gene expression profiles from multiple data cohorts, the R package harmony (109) was used to correct the batch effect.

Based on the batch-effect-corrected expression profile and the best PCs, the “FindNeighbors” and “FindClusters” functions were used to perform unsupervised clustering of cells. The clustering results were visualized using the Uniform Manifold Approximation and Projection (UMAP) (110) and t-distributed Stochastic Neighbor Embedding (tSNE) (111) methods.

### Annotation of cell clusters

The following four methods were used to comprehensively annotate cell clusters to improve the bias that may be introduced by a single annotation method: 1. Use the function “FindAllMarkers” in the R package Seurat and set the parameter “only.pos” to True to identify the significantly (adjusted p<0.01) upregulated differentially expressed genes in each cell cluster. Subsequently, these genes were sorted from largest to smallest by average log2FC within each cell cluster, and 10 genes were grouped into a gene set and entered into the “Cell Type Predictor” in Cell Taxonomy (112) to predict the cell type of each cell cluster. When the predicted cell types had the same score, the second gene set was added, and so on, until the unique highest-scoring cell-type annotation was obtained. 2. Use the R package SingleR (113) to perform an automated cell type annotation process based on two background datasets: HumanPrimaryCellAtlas (HPCA) (114) and BlueprintEncode (BPE) (115, 116). 3. Download the marker genes of all cell types from the Cell Marker (117) database, and then use the R package AUCell (118) to compute the gene set score of each cell cluster. The predicted cell type of each cell cluster was defined as the cell type with the highest score. 4. In the original data cohorts, there are 23,924 cells with known cell type labels, which are used as an auxiliary clue for the annotation of each cell cluster.

Finally, the cell cluster annotation results from the four methods above are integrated, and the final cell type label for each cell cluster is determined by manual verification.

### Identification and phenotypic annotation of cancer-associated fibroblasts

Cells assigned to the broad fibroblast compartment during major-cell-type annotation were extracted from the integrated OSCC single-cell atlas and independently reclustered. Cluster-specific differentially expressed genes were identified using the FindAllMarkers function in Seurat, with positively expressed genes ranked according to their average log2 fold changes. The top-ranked differentially expressed genes from each cluster were submitted to the Cell Type Predictor in Cell Taxonomy (112) for refined cell-type annotation.

The first round of reclustering and annotation was used to refine the fibroblast compartment and remove contaminating populations. The retained CAFs were subsequently reclustered to characterize their internal heterogeneity. Cluster-specific differentially expressed genes were again identified using FindAllMarkers, ranked according to their average log2 fold changes, and submitted to the Cell Type Predictor in Cell Taxonomy for a second round of phenotypic annotation.

### Identification of OSCC malignant cells

Copy number variation (CNV) is a common genomic feature of malignant tumors and contributes to tumor initiation and progression (119). As OSCC originates from oral mucosal epithelial cells (120), malignant epithelial populations were inferred from the epithelial-cell compartment using the R package inferCNV (121). Endothelial cells were selected as the putative non-malignant reference population because they represent a non-epithelial stromal lineage, and their use as the sole reference population for inferCNV analysis has precedent in single-cell studies of head and neck squamous cell carcinoma (122). Large-scale chromosome-level CNV profiles were inferred for each epithelial-cell cluster relative to the endothelial reference cells. Epithelial-cell clusters with significantly higher CNV scores than the reference endothelial cells were classified as putative OSCC malignant epithelial-cell clusters using a two-sided Wilcoxon rank-sum test (P<0.05).

### Identification of ITH expression metaprogram

The ITH expression program refers to a gene set with coordinated expression characteristics in malignant cells. ITH expression programs reveal multiple cellular dysregulation states that effectively reflect the ITH of a malignant tumor (123–125). Previous studies reported ITH expression metaprograms (MPs) in various tumors, which represent the consensus of ITH expression programs across different individual tumors, and explained that MPs are the core source of ITH (9, 36).

Therefore, in our study, to further explore the ITH of OSCC, MPs were identified and defined in OSCC malignant cells as follows: 1. Identify ITH expression programs of each tumor. Only 17 OSCC samples with at least 100 malignant cells were retained. Subsequently, based on the expression profile of HVGs, the R package NMF (126) was used to perform nonnegative matrix factorization (NMF) analysis on the malignant cells from each sample, with the parameter “k” set to 5-10. The optimal k value was selected based on the cophenetic correlation coefficient (127). The function “extractFeatures” was used to extract the top 50 genes with the highest contribution scores to describe each ITH expression program. 2. Identify MP. The Jaccard index between all ITH expression programs was calculated, i.e., the ratio of the intersection to the union of the top 50 genes. Subsequently, using the Jaccard index matrix, the function “cutree” was applied to perform unsupervised hierarchical clustering to identify consensus programs (MPs) across all ITH expression programs. 3. Define MPs. In each MP, genes with a sharing rate of ≥75% across member ITH expression programs were extracted as the MP’s marker genes. Then, the function “enrichGO” in the R package clusterProfiler (128) was used to perform gene ontology (GO) (129) enrichment analysis based on the above marker genes of each MP. GO terms with adjusted p<0.05 and q<0.05 were considered statistically significant. Finally, the biological function of each MP was defined by the GO terms with the top 5 smallest adjusted p-values and their marker genes.

### Spatial feature analysis of MPs

To reveal the spatial feature of MPs’ expression, we used the R package Seurat (version 4) to perform the standard 10X Visium spatial transcriptome analysis pipeline. First, the functions “SCTransform” and “FindSpatiallyVariableFeatures” were used to standardize gene expression across spots and identify spatial HVGs, which effectively reflect gene expression characteristics related to spatial location (21). Then, dimensionality reduction and unsupervised clustering analysis were performed on all spots to generate spot clusters. These spot clusters were mapped to the corresponding pathological slice images. Based on pathological knowledge, these spot clusters were merged, and finally, all spot were divided into three pathological regions: tumor core (TC), tumor front (TF), and tumor periphery (TP).

Based on the marker genes of each MP, the MP expression scores of spots were calculated using the function “AddModuleScore” and mapped to the pathological regions. The R packages ggpubr (130) and gghalves (131) were used to draw grouped box plots to quantify the expression level of each MP in three pathological regions. Differences in MP scores and deconvolved cell-subcluster scores between pathological regions were evaluated using two-sided Wilcoxon rank-sum tests. The corresponding P values are indicated in the relevant figures.

### Construction of the dynamic exhaustion evolution trajectory of CD8+ T cells

Low T cell receptor (TCR) diversity and high clonal expansion are the main characteristics of exhausted T cells. The diversity and scale of T cell receptor (TCR) clones can effectively reflect the exhaustion level of T cells (17). In our study, to accurately reveal the dynamic exhaustion mechanism of CD8+T cells in the OSCC TME, we first applied the standard unsupervised clustering analysis pipeline in the R package Seurat to scRNA-Seq data on T cells to identify CD8+ T cell subtypes. Then, CD8+ T cells with paired scTCR-Seq data were extracted for the next analysis.

The R package Monocle3 (132) was used to analyze pseudotime trajectories of CD8+ T cells. The CD8+ T cells were clustered using the functions “reduce_dimension” and “cluster_cells”. Then, the functions “learn_graph” and “order_cells” were used to identify the gene expression changes of each cell based on the UMAP, thereby inferring the cell trajectory. The pseudotime values were assigned to each cell based on its position along the trajectory to quantify each cell’s temporal state. On the other hand, the paired scTCR-Seq data were imported and integrated into the monocle3 object, and the frequency of all TCR clonal types was counted and mapped to the cell trajectory using the function “plot_cells” to represent the scale of CD8+ T cell clonal expansion.

Our study confirmed and verified the validity and accuracy of the CD8+ T cell exhaustion trajectory from the following three aspects: 1. Exhaustion phenotype marker genes (LAG3, PDCD1, TIGIT, HAVCR2) and pre-dysfunction marker genes (GZMK, GZMA) were obtained from known study (133), and the expression trend of the above genes over pseudotime was visualized using the function “plot_genes_in_pseudotime”. 2. To confirm whether pseudotime can accurately reflect the clonal expansion scale of CD8+ T cells, the correlation analysis between the clonal expansion scale of CD8+ T cells and pseudotime values was performed. First, the Pearson correlation coefficient was calculated using the function “cor.test”, and then the correlation curve was fitted using the function “geom_smooth”. 3. The CD8+ T cell subclusters were mapped to the cell trajectory to evaluate the distribution characteristics.

### Spatiotemporal analysis of CD8+ T cell exhaustion phenotype

Based on the quartiles of the pseudotime distribution, CD8+ T cells were divided into four ordered intervals, termed levels 1–4, corresponding to the 0–25%, 25–50%, 50–75%, and 75–100% ranges of pseudotime, respectively. These levels were used as reference intervals for spatial deconvolution and were not interpreted as discrete biological subtypes. Paired scTCR-Seq clone sizes were mapped onto the trajectory and correlated with pseudotime to support its biological interpretation. Because the spatial analysis required estimation of the relative contributions of predefined single-cell-derived transcriptional states within mixed Visium spots, CIBERSORT was selected as a reference-based deconvolution method that could be applied consistently to both the four pseudotime intervals and the annotated T cell subclusters. The mean gene-expression profile of each interval was then used as a reference signature, and CIBERSORT (134) was applied to estimate the proportions of levels 1–4 in each spatial spot.

### Analysis of spatiotemporal interactions of T cells

Using the same CIBERSORT-based framework, the relative proportion of each T cell subcluster was estimated in each spatial spot and mapped to the pathological regions.

To obtain a continuous summary of the inferred CD8+ T cell trajectory in each spatial spot, we calculated a pseudotime-weighted score, T_spot_, as follows:


Tspot=Plevel1∗1+Plevel2∗2+Plevel3∗3+Plevel4∗4

ZXXCXThe P_level 1–4_ represent the estimated proportions of the four pseudotime intervals in each spot. Since exhaustion subtypes have larger pseudotime values, T_spot_ can reflect the overall pseudotime and exhaustion level of each heterogeneous spot. Its relationships with the estimated abundance of T cell subtypes were subsequently evaluated using correlation and curve-fitting analysis.

### Cellular communication analysis

To explore the role of CAFs in the OSCC TME, the R package CellChat (135) and the Python package CellPhoneDB (136) were used to analyze the intercellular communication between CAFs and other cell subclusters.

Since most non-protein signals are specific synaptic signal interactions and can only be used to infer communication between neurons (135), the function “subsetDB” was first used to remove non-protein signal interactions from the CellChatDB.human background. Then, the function “computeCommunProb” was used to compute the interaction strength between each pair of cell subclusters. The parameter “type = triMean” was set to retain more robust interaction relationships. The function “aggregateNet” was used to construct the communication network. Finally, the function “netVisual_circle” was used to visualize the number of interaction pairs and the interaction strength. To further uncover the communication mechanism between well-interaction cell subclusters, the analysis was performed at the gene and signal pathway levels, and the results were visualized using the functions “netVisual_chord_gene” and “netAnalysis_signalingRole_scatter”.

On the other hand, CellPhoneDB (version 5) was used to analyze intercellular communication based on receptor-ligand interactions, further verifying the results from CellChat. Our study adopted the statistical analysis pipeline (136) and counted only receptor-ligand interaction pairs with adjusted p< 0.05 to quantify the communication strength between each cell subcluster.

### Spatial co-localization analysis

The position, proximity, and arrangement of cells in tissues directly affect the intensity, range, and effectiveness of signal transmission (137–139). Based on this, we defined the spatial similarity to quantify the strength of spatial co-localization between cells.

Spatial similarity was used to quantify the spatial colocalization of two cell subclusters by integrating their deconvolved abundances across spots with the Euclidean distances between those spots. Higher spatial-similarity values indicate that the two subclusters are more abundant in spatially proximal spots, whereas lower values indicate weaker or more spatially separated co-occurrence. The calculation formula for the spatial similarity S_ij_ between cell subcluster i and cell subcluster j is as follows:

$\text{Sij}=\{\begin{array}{cc} 0 & \text{dist}\left(\text{a,b}\right)>3\\ \text{Cai}*\text{Cbj}*\frac{1}{\text{dist}\left(\text{a,b}\right)+1} & \text{dist}\left(\text{a,b}\right)<=3 \end{array}$C_ai_ and C_bj_ represent the abundance of cell subcluster i in spot a and the abundance of cell subcluster j in spot b, respectively. The abundance is calculated using the R package spacexr (140) based on the robust cell type decomposition (RCTD) algorithm. Specifically, the gene expression profile and cell subcluster labels were used as input to the function “Reference” to construct a reference background. Then, the spatial coordinate information and gene expression values for spots were passed to the function “SpatialRNA” to construct a spatial object. Finally, the calculation is run using the functions “create.RCTD” and “run.RCTD”.

The calculation formula of the Euclidean distance between spot a and spot b, dist (a, b), is as follows:

$\text{dist}\left(\text{a,b}\right)=\frac{\sqrt{{\left({\text{x}}_{\text{a}}-{\text{x}}_{\text{b}}\right)}^{2}+{\left({\text{y}}_{\text{a}}-{\text{y}}_{\text{b}}\right)}^{2}}}{\text{fiducial\_diameter\_fullres}}$x_a_, y_a_, x_b_, y_b_ represent the horizontal and vertical coordinate values of spot a and spot b, respectively, and fiducial_diameter_fullres is used to describe the diameter of spot in high-resolution images.

### Spatial interaction analysis

Spatial interaction analysis were performed separately for the GSM5494476 and PRJNA811533 spatial transcriptomic datasets using stLearn. Spot-level CAF and myeloid subcluster scores obtained from spatial deconvolution were aligned with the corresponding spatial barcodes and integrated with the spatial gene-expression matrices.

Genes detected in fewer than three spots were excluded. Human ligand–receptor pairs were obtained from the connectomeDB2020_lit database. Spatially significant ligand–receptor hotspots were identified using the spatially adjacent spot network, with a minimum of 10 supporting spots and 10,000 randomly generated background pairs. The resulting P values were adjusted using the Benjamini–Hochberg method, and ligand–receptor hotspots with an adjusted P value<0.05 were considered significant.

Directional cell–cell interactions from PI3+ CAF to individual myeloid subclusters were subsequently evaluated using the mixed-spot cell–cell interaction framework. A subtype score cutoff of 0.20 was used to define the presence of a cell subcluster within a spot. Interactions supported by at least three spots were tested using 1,000 permutations, and P<0.05 was considered statistically significant.

To visualize the spatial distribution of ligand–receptor signals supporting a specific directional cell pair, we constructed a CCI-weighted ligand–receptor hotspot score. For each ligand–receptor pair, its significant spatial hotspot score was divided by the maximum score across spots and then weighted by the corresponding significant cell–cell interaction count for that directional pair. The weighted scores were averaged across all significant ligand–receptor pairs.

### Multiplex immunofluorescence assay

Paraffin-embedded tissues were cut into 5µm sections and attached to poly-lysine-treated slides. The slices were dried at 60 °C for 2–8 hours and dewaxed with xylene for 30 minutes. Gradient alcohol was used for rehydration and antigen retrieval. PBS was used for 3 minutes. Serum was added, and the plate was blocked in a humidified box at 37 °C for 30 minutes. The blocking solution was wiped off with filter paper, and the primary antibodies (COL1A1: 67288-1-Ig, Proteintech; PI3: DF14564, Affinity) at appropriate concentrations were added and incubated in a humidified box at 4 °C overnight. The primary antibody was washed off with PBS for 3 minutes for 5 times. Fluorescent secondary antibody (S0B4007, STARTER; S0B4017, STARTER) was added and incubated in a humidified box at room temperature for 1 hour in the dark. The secondary antibody was washed off with PBS for 3 minutes for 3 times. DAPI was added and incubated for 2 minutes in the dark to visualize tissue nuclei. DAPI was washed off with PBS for 1 minute for 3 times. Finally, the slides were mounted in glycerol and imaged using confocal laser microscopy (Cat#0972818, Olympus, Japan). Images were processed using ZEISS ZEN (version 3.11) software.

### Identification of OSCC ecotypes

OSCC ecotypes were defined as patient groups with similar inferred cell-subcluster compositions. In contrast to the basal, mesenchymal, atypical, and traditional molecular subtypes derived from bulk gene-expression patterns, ecotypes were designed to describe patient-level ecosystem composition and provide cellular interpretability rather than replace the established molecular classification.

First, the CIBERSORT deconvolution algorithm was used to resolve the cell subcluster composition in each bulk sample and obtain the cell subcluster composition matrix. The background matrix used for deconvolution was derived from the mean expression values of the union (16,638 genes after deduplication) of significantly upregulated differentially expressed genes in 63 cell subclusters. Then, the NMF unsupervised clustering algorithm was used to perform column clustering on the cell subcluster composition matrix, with the parameter “k” set to 2-10, and the optimal k value was selected based on the cophenetic correlation coefficient.

### Identification of OSCC coordinated cell program

Following the framework proposed by Chen et al. (66), CCPs were defined as groups of cell subclusters with coordinated abundance patterns across patients. Whereas ecotype clustering was performed across the patient dimension of the composition matrix, CCP clustering was performed across the cell-subcluster dimension to identify cellular co-enrichment patterns.

First, the NMF unsupervised clustering algorithm was used to perform row clustering on the cell subcluster composition matrix, with the parameter “k” set to 2-10, and the optimal k value was selected based on the cophenetic correlation coefficient. The enrichment level of CCPs in each OSCC ecotype was visualized, and the Wilcoxon rank-sum test was used to assess statistical significance.

### Association analysis between OSCC ecotypes and clinical characteristics

Our study performed a detailed association analysis between OSCC ecotypes and clinical characteristics from 11 aspects, including OSCC traditional molecular subtypes, prognosis, recurrence rate, HPV infection, lifestyle habits (including smoking and drinking history), immunotherapy response, age, gender, tumor grade, and clinical stage.

Among the 1017 patients, only 179 (from the GSE65858) had OSCC traditional subtype labels. Therefore, to assign traditional subtype labels to all patients, we first constructed a traditional subtype background matrix using the gene expression profiles of the 179 patients above. Then, the abundance of traditional subtypes of each patient was predicted using the CIBERSORT deconvolution algorithm. Finally, the traditional subtype with the highest abundance was assigned to each patient. The R package treemap (141) was used to draw a rectangular tree structure diagram to intuitively visualize the distribution and proportion of traditional subtypes in each OSCC ecotype.

For prognosis analysis, the R packages survival (142) and survminer (143) were used to plot stratified Kaplan-Meier (KM) (144) survival curves to reveal the prognostic differences between patients with different OSCC ecotypes. The statistical significance of the differences was tested using the log-rank test (145).

For binary or multi-class classification of discrete clinical characteristics (including recurrence, HPV infection, gender, smoking history, drinking history, clinical stage, tumor grade, and immunotherapy response), patients with the above clinical information were collected, respectively. Then, the frequency was counted according to the OSCC ecotype label, with smoking history, drinking history, and recurrence all counted as “yes”; HPV infection as “positive”; anti-PD-1 immunotherapy response as “response”; and gender as “female”. The significance of the association between OSCC ecotype and the above clinical characteristics was assessed using the chi-square test in R with the “chisq.test” function. Since not all patients had the true immunotherapy response labels, the tumor immune dysfunction and exclusion (TIDE) (146) was used to predict the response of all patients to anti-PD-1 immunotherapy based on their gene expression profiles.

For continuous clinical characteristics such as age, box plots were constructed using OSCC ecotype labels, and the Wilcoxon rank-sum test was used to assess differences between groups.

### Prediction of drug sensitivity

The function “calcPhenotype” in the R package oncoPredict (147) was used to predict the chemotherapy drug sensitivity of all 1017 patients. The parameters “trainingExprData”, “trainingPtype”, and “testExprData” corresponded to the gene expression profile of the OSCC cell lines, the drug sensitivity matrix of the OSCC cell lines, and the gene expression profile of all 1017 patients, respectively. Drug sensitivity was expressed as the AUC, with smaller values indicating higher drug sensitivity (148).

To improve the current status of OSCC treatment, we further identified OSCC ecotype-specific candidate drugs (or compounds) for patients with ecotype I and ecotype III who are insensitive to current first-line chemotherapy drugs, respectively. First, the drug sensitivity of all 1017 patients was predicted. Then, OSCC ecotype-specific candidate drugs were identified according to the following steps: 1. Calculate the mean AUC of each drug in patients from a given ecotype, denoted as S_mean_, and then in each cohort (GDSC1, GDSC2, CTRP2, and PRISM), identify the drugs that exhibit the minimum S_mean_ in a given ecotype, denoted as D_mean_. 2. Calculate the median AUC of each drug in patients from a given ecotype, denoted as S_median_, and then in each cohort, identify the drugs that exhibit the minimum S_median_ in a given ecotype, denoted as D_median_. 3. In each cohort, retain the union of D_mean_ and D_median_, and record these drugs as D_candidate_, respectively. 4. Only retain D_candidate_ that appear in two or more cohorts. 5. For the above D_candidate_, use the ecotype of patients as the grouping label, draw a drug sensitivity grouping box plot, and evaluate the significance of the difference between groups by the Wilcoxon statistical test, and only retain drugs with p<0.05 for manual verification.

### Construction and validation of the prognostic risk prediction model based on OSCC ecology

To construct the prognostic risk prediction model, a submatrix comprising 658 patients with available survival information was extracted from the patient-by-cell-subcluster composition matrix. Univariable Cox regression was first performed separately for each cell-subcluster abundance using the coxph function in the R package survival. Cell subclusters associated with survival at P<0.05 were retained as candidate predictors. These candidate predictors were subsequently entered into a LASSO-penalized model implemented using the R package glmnet (149). The LASSO (150) penalty was used to shrink model coefficients and perform variable selection by reducing the coefficients of less informative predictors to zero. Ten-fold cross-validation was performed using cv.glmnet to select the regularization parameter λ. The final model was constructed at lambda.min, and cell subclusters with non-zero coefficients were retained as model predictors.

To assess the model’s predictive efficacy, both internal and external validation were performed. For internal validation, the training samples were first risk-scored using the model. Then, the samples were automatically grouped into high-risk and low-risk based on the risk score using the functions “surv_cutpoint” and “surv_categorize” in the R package survminer. The function “ggsurvplot” was used to plot the KM survival curve, and the prognostic difference between the two groups was tested using the log-rank test. For external validation, five external OSCC data cohorts with prognostic information were collected from the GEO database (accession numbers: GSE75538 (151), GSE75539 (151), GSE85446 (152), GSE111390 (153), and GSE117973 (154)). Only primary OSCC samples with sampling sites in the oral cavity were retained. The data cohorts GSE75538, GSE75539, GSE111390, and GSE117973 were merged to mitigate bias arising from insufficient observations. Finally, three data cohorts were generated: GSE75538+GSE75539 (20 samples), GSE85446 (66 samples), and GSE111390+GSE117973 (64 samples) for external validation of the model. The external validation pipeline was consistent with the internal validation pipeline.

### Construction and validation of the recurrence risk prediction model based on OSCC ecology

Recurrence is one of the main reasons for OSCC treatment failure (155–157). Therefore, we further explored whether the cell subcluster composition of OSCC could predict recurrence risk in patients.

Among the 1,017 patients, recurrence information was available for only 134 patients. Given the limited sample size relative to the number of candidate cell-subcluster predictors, a ridge-penalized binary logistic regression model (158) was constructed using the R package glmnet. Unlike LASSO, ridge regression was used primarily to shrink the coefficients of correlated or weakly informative predictors toward zero rather than to perform sparse variable selection. Ten-fold cross-validation was used to select the regularization parameter λ, and the resulting ridge coefficients were used to calculate the recurrence-risk score. The R package pROC (159) was used to fit the receiver operating characteristic (ROC) curve of the model, and the AUC was calculated to evaluate the model’s predictive performance on internal training data.

To verify the robustness of the model, external OSCC data cohorts with recurrence information were collected from the GEO database (accession numbers: GSE75538 (151), GSE75539 (151), and GSE87593 (160)). Data cohorts GSE75538 and GSE75539 contain disease-free survival (DFS) information. We performed external data validation of the recurrence risk prediction model from the following two aspects: 1. After merging, the data cohorts GSE75538 and GSE75539 contained a total of 14 samples with DFS information for more than one year after the first treatment. Then, the CIBERSORT deconvolution algorithm was used to infer the cell subcluster composition of all samples, and based on this, the recurrence risk score was calculated through the prediction model. All samples were divided into a high-recurrence-risk group and a low-recurrence-risk group using the functions “surv_cutpoint” and “surv_categorize” based on the recurrence risk score. Finally, the KM survival curve was drawn to reveal the DFS difference between the groups. 2. The data cohort GSE87593 contains a total of 8 samples with recurrence labels, including 4 recurrence samples and 4 non-recurrence samples. First, the CIBERSORT deconvolution algorithm was used to infer the cell subcluster composition of all samples. Then, the prediction model was used to score the recurrence risk of samples. Using the true and predictive recurrence labels, the ROC curve of the prediction model was fitted, and the AUC was calculated to evaluate the model’s accuracy and robustness for binary classification.

### Construction and validation of the anti-PD-1 immunotherapy sensitivity prediction model based on OSCC ecology

Using the TIDE-inferred immunotherapy-response labels and the patient-by-cell-subcluster composition matrix of 1,017 patients, a LASSO-penalized binary logistic regression model was constructed using the R package glmnet. Ten-fold cross-validation was used to select the regularization parameter λ, and the retained predictors and their corresponding coefficients were used to calculate the immunotherapy-sensitivity score. Model discrimination in the development cohort was evaluated using the receiver operating characteristic curve and the area under the curve.

On the other hand, external data cohorts were used to further verify the robustness of the model from two aspects: 1. Since the score calculated by the model represents the immunotherapy sensitivity of samples and the TIDE score represents the level of immune escape of samples, if the model score is negatively correlated with the TIDE score, it can prove that the model has good robustness. Based on this, 6 external OSCC data cohorts were collected from the GEO database, including 5 cohorts that have already been used to validate the prognostic risk prediction model and GSE142583 (161). First, the cell subcluster composition of all samples was inferred by the CIBERSORT deconvolution algorithm. Then the model was used to score. At the same time, TIDE scores for all samples from the 6 data cohorts above were calculated using the TIDE online tool. Finally, in each data cohort, the correlation and significance between the TIDE score and the model score were evaluated using the function “cor.test”. 2. Although anti-PD-1 immunotherapy has been widely used in melanoma, OSCC data cohorts with immunotherapy response labels are still relatively scarce, and the response mechanism of immunotherapy in different tumors is similar (162). Therefore, we obtained a melanoma data cohort with true immunotherapy response labels from GSE78220 (163) to evaluate the model’s predictive performance. The CIBERSORT algorithm was used to infer the cell subcluster composition of all samples, and then the model was used to score. Based on the true and predictive immunotherapy response labels, the ROC curve was fitted, and the AUC was calculated. In addition, the grouped box plot was drawn with immunotherapy response as the grouping variable to intuitively reveal differences in model scores between the response and non-response groups.

### Construction of OSCC Atlas web server

The OSCC Atlas web server was built based on the R package shiny (164). The R package shinydashboard (165) was used to control the web page layout. OSCC Atlas mainly consists of two modules: “Browse” and “Tool”.

The Browse module includes the OSCC cell atlas’s visualization function. All graphics are drawn in real time using the R package Seurat and rendered in the user interface, while allowing users to download them as PNG files. Specifically, the dimensionality reduction map of unsupervised cell clustering is generated using the function “DimPlot”, with the parameters “object” and “reduction” set to the selected cell type and dimensionality reduction method, respectively. The violin plot of gene expression is drawn using the function “VlnPlot”, with the parameters “object” and “features” set to the selected cell type and the gene name entered by the user, respectively. The dimensionality reduction mapping plot of gene expression is drawn using the function “FeaturePlot”, with the parameters “object”, “features”, and “reduction” set to the selected cell type, the entered gene name, and the user-selected dimensionality reduction method, respectively.

The Tool module provides an online analysis platform that allows users to upload bulk RNA-Seq data from an OSCC patient to evaluate the cell subcluster composition in tumor tissue and predict the patient’s OSCC ecotype. More importantly, through online model scoring, users can intuitively evaluate the patient’s prognosis risk, recurrence risk, and immunotherapy sensitivity, thereby assisting clinical treatment decision-making. When the user uploads the data and clicks the submit button, the function “read.table” is first used to read the uploaded data and verify its format. To facilitate the user in confirming the uploaded data, the function “renderTable” is used to render a table on the web page, displaying the original data uploaded by the user. Then, the CIBERSORT algorithm is used to infer the tissue’s cell subcluster composition from the gene expression profile uploaded by the user. The result is visualized in two forms: 1. The data table allows users to download in txt format. 2. The word cloud graphics are drawn based on the R package wordcloud2 (166) and rendered to the web page in real time through the function “renderWordcloud2”. Users can also download the graphics in PNG format.

To predict the OSCC ecotype and clinical characteristics of the patient, the following two analysis were performed based on the cell subcluster composition matrix: 1. We first constructed a four-classification LASSO generalized linear model for OSCC ecotype prediction and embedded it in the web server for online prediction. Under this model, each patient will receive 4 scores, representing the weights of the 4 OSCC ecotypes, with the highest-weighted OSCC ecotype as the predicted ecotype. To facilitate users’ intuitive quantification of the ecotype to which the patient belongs, the four model outputs are standardized to be between 0 and 1, with a sum of 1, representing the probability that the patient belongs to each OSCC ecotype. 2. The patient’s prognostic risk, recurrence risk, and immunotherapy sensitivity are scored respectively and rendered in a table on the web page. To facilitate users’ more intuitive evaluation of the above three clinical characteristics of the patient, the scores of the training samples were used as the background, and five segments were defined based on the background range, namely, lowest, low, middle, high, and highest. On the web page, a colored bar diagram is drawn using the R package shinyjs (167) to represent the background, and the current patient’s score is indicated by an arrow. The definition of the above five segments is as follows:

$\text{P}=\frac{\text{model score}-\text{the minimum score of background}}{\text{the maximum score of background}-\text{the minimum score of background}}*100$

When P<=20, it is defined as Lowest; when 20<P<=40, it is defined as Low; when 40<P<=60, it is defined as Middle; when 60<P<=80, it is defined as High; when P>80, it is defined as Highest.

To improve the stability and performance of the web server, all online analysis are run through the R package future (168), using a parallel computing framework with a multisession strategy.

### Statistical analysis

Unless otherwise specified, all operations in our study were performed using R language software (version 4.2). The Wilcoxon test was used to analyze the differences between the groups, the log-rank test was used to analyze the differences in prognosis, and the Pearson correlation was used to measure the correlation between samples. P< 0.05 was considered statistically significant.

## Discussion

Despite the availability of multiple treatment options for OSCC in clinical practice, a significant number of patients experience local recurrence after initial therapy, and emerging immunotherapies show promising responses in only a small subset of OSCC patients. The complex heterogeneity of OSCC is a key factor behind these clinical challenges. However, current studies typically analyze the intratumor heterogeneity (ITH) of OSCC based on single-omics data or by focusing on only one cell type, which leads to a biased view. Therefore, considering the current clinical landscape and the limitations of previous research, we thoroughly investigate the complex heterogeneity and personalized medicine strategies for OSCC from a comprehensive tumor ecosystem perspective, utilizing multi-omics cohorts.

Based on multi-omics data, we built a multidimensional OSCC cellular atlas that includes 88,442 cells. This atlas features five main cell types—malignant cells, T cells, B cells, myeloid cells, and cancer-associated fibroblasts (CAFs)—along with 63 cell subclusters. The biological differences among malignant cell subclusters and expression meta-programs (MPs) revealed the complex ITH of OSCC from two angles. More importantly, the combined analysis of scRNA-Seq and ST-Seq data showed that, compared to other malignant MPs, the fibroblast proliferation MP was significantly upregulated at the tumor front regions of OSCC. Previous single-cell work by Puram et al. characterized malignant and stromal heterogeneity within HNSCC tumor ecosystems (30). Building on this context, our integrated spatial and cell–cell communication analysis identified a reproducible association between PI3+ CAF and APC_Myeloid across two OSCC sections and nominated a potential collagen-associated communication axis. The contribution is therefore the identification of a specific stromal–myeloid relationship within this heterogeneous ecosystem. However, spatial association and inferred ligand–receptor interactions do not demonstrate that PI3+ CAF impairs antigen presentation; this proposed functional consequence remains to be tested experimentally. Previous single-cell studies have characterized heterogeneous tumor-infiltrating T-cell populations in OSCC (87), while established exhaustion frameworks describe the acquisition of dysfunction-associated states under persistent antigen stimulation (15, 169). Our findings are consistent with these frameworks and add an integrated view linking CD8+ T-cell transcriptional states to TCR clonal expansion and their inferred spatial distributions. In particular, the association of intermediate pseudotime states with Treg- and inflammatory NKT-cell-rich tumor-front regions suggests a spatial context for exhaustion-associated changes. However, these cross-sectional analysis do not establish a temporal differentiation sequence, tumor-antigen specificity, or a causal role for neighboring immune cells in inducing exhaustion.

To characterize OSCC ecosystem organization, we identified four OSCC ecotypes and four coordinated cell programs (CCPs) from complementary dimensions of the patient-by-cell-subcluster composition matrix. Ecotypes grouped patients with similar inferred cellular compositions, whereas CCPs grouped cell subclusters with coordinated abundance patterns across patients. Earlier HNSCC classifications by Chung et al. and Walter et al. defined molecular subtypes from bulk gene-expression profiles (7, 8). Our framework complements these classifications by describing tumors through inferred cellular composition and identifying cell subclusters that co-vary across patients. The associations between our ecotypes and established molecular subtypes indicate overlapping biological features, while the CCPs provide a cellular interpretation of these ecosystem patterns. Because CCP derivation follows the framework of Chen et al. (66), the contribution lies in its application to OSCC and the resulting cellular associations, rather than the introduction of a new clustering method. Whether this framework adds predictive value beyond established molecular subtypes remains to be determined. Based on this, we explored precision medication strategies and built prediction models for three clinical features. Compared to traditional gene-based prediction models, our models based on cell composition can directly reflect the impact of relevant subclusters on specific clinical traits through coefficients. This approach not only enables a deeper understanding of the biological functions of specific subclusters during OSCC initiation and progression but also clearly shows the intrinsic relationship between the OSCC microenvironment and clinical phenotypes. We also developed the OSCC Atlas web platform to facilitate exploration of the cellular atlas, ecosystem classifications, and associated analytical models.

Overall, the multi-omics atlas developed in this study provides an integrated view of malignant, stromal, and immune-cell heterogeneity and their interactions within the OSCC microenvironment. By combining cellular-state characterization, spatial organization, immune-repertoire dynamics, ecological stratification, and clinical modeling, this study provides a framework for investigating OSCC as a complex multicellular ecosystem and for generating testable hypotheses regarding potential therapeutic vulnerabilities. However, these findings should be interpreted in the context of the exploratory design of the study. The analysis were largely based on retrospectively collected public datasets, including a limited number of spatial transcriptomic samples and bulk cohorts with heterogeneous clinical and technical characteristics, which may affect the generalizability of the observed spatial, ecological, and clinical associations. In addition, several conclusions—including malignant-cell classification based on inferred CNV profiles, cell–cell communication, ecotype assignment, clinical prediction, and drug-response prioritization—were derived primarily from computational analysis and therefore require further genomic, functional, and clinical validation. In particular, the proposed ecotypes and prediction models should not yet be regarded as established clinical classification or decision-support tools, and the drug associations derived from cancer cell-line resources should be considered candidates for subsequent experimental evaluation rather than direct treatment recommendations.

Future studies incorporating larger and clinically well-annotated spatial and bulk cohorts, additional molecular modalities, functional perturbation experiments, and prospective validation will be required to refine the proposed OSCC ecosystem framework and determine its potential value for patient stratification and therapeutic development.

## Data Availability

The original contributions presented in the study are included in the article/**Supplementary Material**. Further inquiries can be directed to the corresponding author.
